# Supplementary material for: Chromatin information content landscapes inform transcription factor and DNA interactions
Source: Nat Commun. 2021 Feb 26;12:1307. doi: 10.1038/s41467-021-21534-4 (PMC7910283; doi:10.1038/s41467-021-21534-4)
Supplement: Supplementary file 1 — Supplementary Information [file 41467_2021_21534_MOESM1_ESM.pdf]

Supplementary Information

Chromatin information content landscapes inform transcription factor and DNA interactions

Ricardo D'Oliveira Albanus, Yasuhiro Kyono, John Hensley, Arushi Varshney, Peter Orchard, Jacob O. Kitzman, Stephen C. J. Parker

Correspondence to: scjp@umich.edu

This PDF file includes:
Supplementary Note
Supplementary Figures 1 to 29
Supplementary Tables 1 and 2
Supplementary References

Supplementary Note

Transcription factor binding prediction methods comparison

BMO builds on previous reports that the degree of chromatin accessibility around a motif[1–3] and the presence of co-occurring motifs[4] positively correlates with TF binding, and uses TF-specific negative binomial models of these two signals to estimate the likelihood of a bound instance (Methods). We benchmarked the performance of BMO and other unsupervised TF binding prediction algorithms using ATAC-seq datasets from the GM12878 and HepG2 cell lines and their corresponding TF ChIP-seq data (n = 41 and n = 59, respectively; Supplementary Data 1). We compared BMO to three footprinting-based algorithms (HINT-ATAC[5], DNase2TF[6], PIQ[7]), to CENTIPEDE[8], which learns informative DNA cut patterns indicating TF binding, and to a baseline classifier that labels TF motifs within ATAC-seq peaks as bound. To evaluate methods, we calculated the area under the precision-recall curve (AUC-PR), which informs the performance of the classifier in ranking bound and unbound motif instances, and the F1 score, which measures the performance of the threshold used to call bound motif instances.

BMO outperformed all methods in our high-signal GM12878 dataset (Fig. 1e), whereas BMO and CENTIPEDE had similarly high performance in lower-signal datasets (Supplementary Fig. 7). DNase2TF had lower performance in the lower-signal datasets (Supplementary Fig. 7). PIQ cannot use custom TF motif scans and therefore required separate benchmarking, which revealed lower performance compared to BMO (Supplementary Fig. 9). These results were consistent across ATAC-seq replicates and cell lines, including downsampled data representing shallower sequencing depths (Supplementary Fig. 10). Of note, the AUC-PR of footprinting-based methods was lower overall due to their inability to classify motifs occurring outside ATAC-seq peaks, which we reason contain true TF binding sites and negatively affect PR-AUCs. While their F1 scores indicate that this effect is less pronounced when taking into account the thresholds to call bound motif instances, their performance was still consistently lower than non footprinting-based methods (Supplementary Fig. 7c). Overall, the footprinting-agnostic methods (BMO and CENTIPEDE) outperformed footprint-based methods on the majority (median of 81% across datasets) of tested TFs. These results indicate that TF binding is more accurately predicted using a simple chromatin accessibility model tuned to each TF motif.

We next sought to determine if the CENTIPEDE approach relied on spatial DNA cut patterns, or if the overall accessibility in the region was sufficient for high performance. We devised an alternative implementation of CENTIPEDE that ignores the DNA cut positions (signal-sum CENTIPEDE; ssCENTIPEDE) and masks any footprint-like patterns, but not the chromatin accessibility in the region (Methods). This ssCENTIPEDE approach ranked motif instances almost identically to CENTIPEDE (Fig. 1e, Supplementary Fig. 11, AUC-PR plots), again indicating that footprint patterns in chromatin profiles are not necessary for high prediction performance. One corollary expectation from this conclusion is that footprint-based algorithms should perform comparatively worse when predicting binding for TFs with a low impact on local chromatin. To test this, we compared performance across f-VICE tertiles representing low (tertile one), intermediate (tertile two), and high (tertile three) f-

VICEs. Notably, BMO and (ss)CENTIPEDE had relatively higher performance on lower f-VICE tertiles one and two (Fig. 1e, Supplementary Fig. 7). Our findings indicate that footprinting-based methods are more sensitive to the local TF-chromatin architecture.

Supplementary Figures 1 to 29

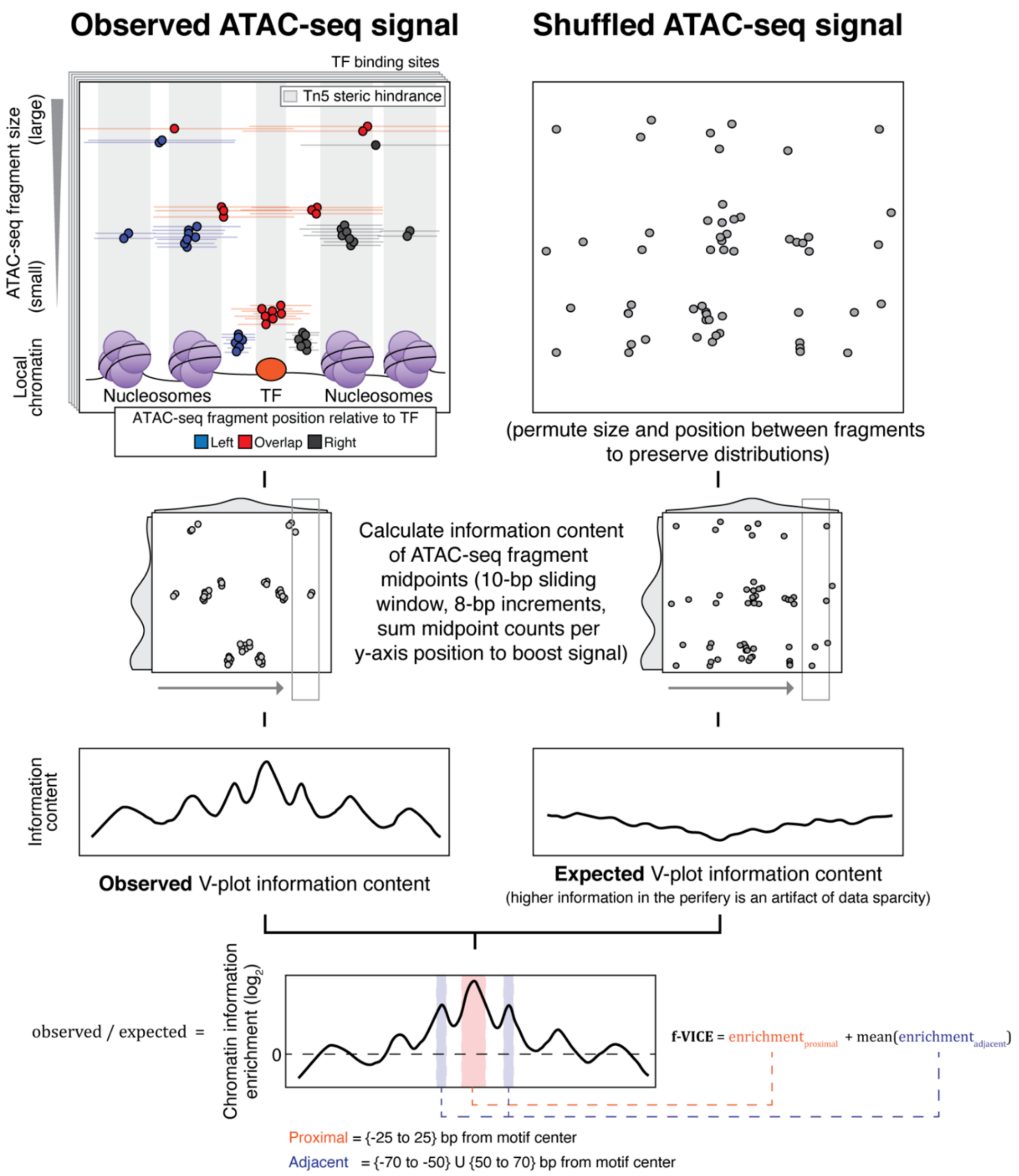

Supplementary Figure 1. Calculating CIE and f-VICEs.
Schematic overview of the CIE and f-VICE calculations.

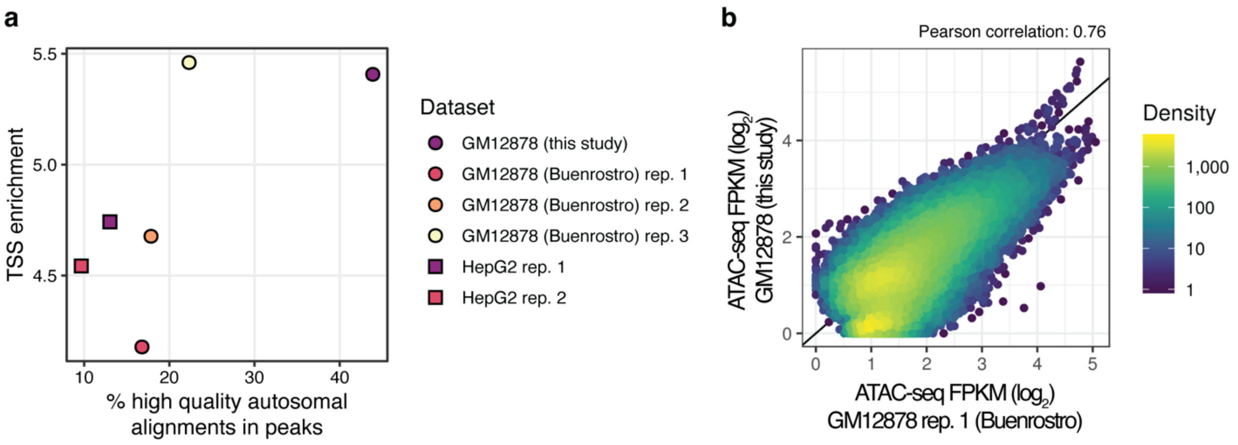

Supplementary Figure 2. ATAC-seq datasets signal-to-noise comparisons.
a Scatter plots of the percent high quality autosomal alignments (%HQAA) in ATAC-seq peaks distribution and TSS enrichments of GM12878 and HepG2 datasets, obtained using Ataqv[9] (github.com/ParkerLab/ataqv). **b** Scatter plot of the ATAC-seq signal in the union of the MACS2 broad peaks called in the two GM12878 datasets. Each point corresponds to one ATAC-seq peak. Solid diagonal line, identity ($x = y$).

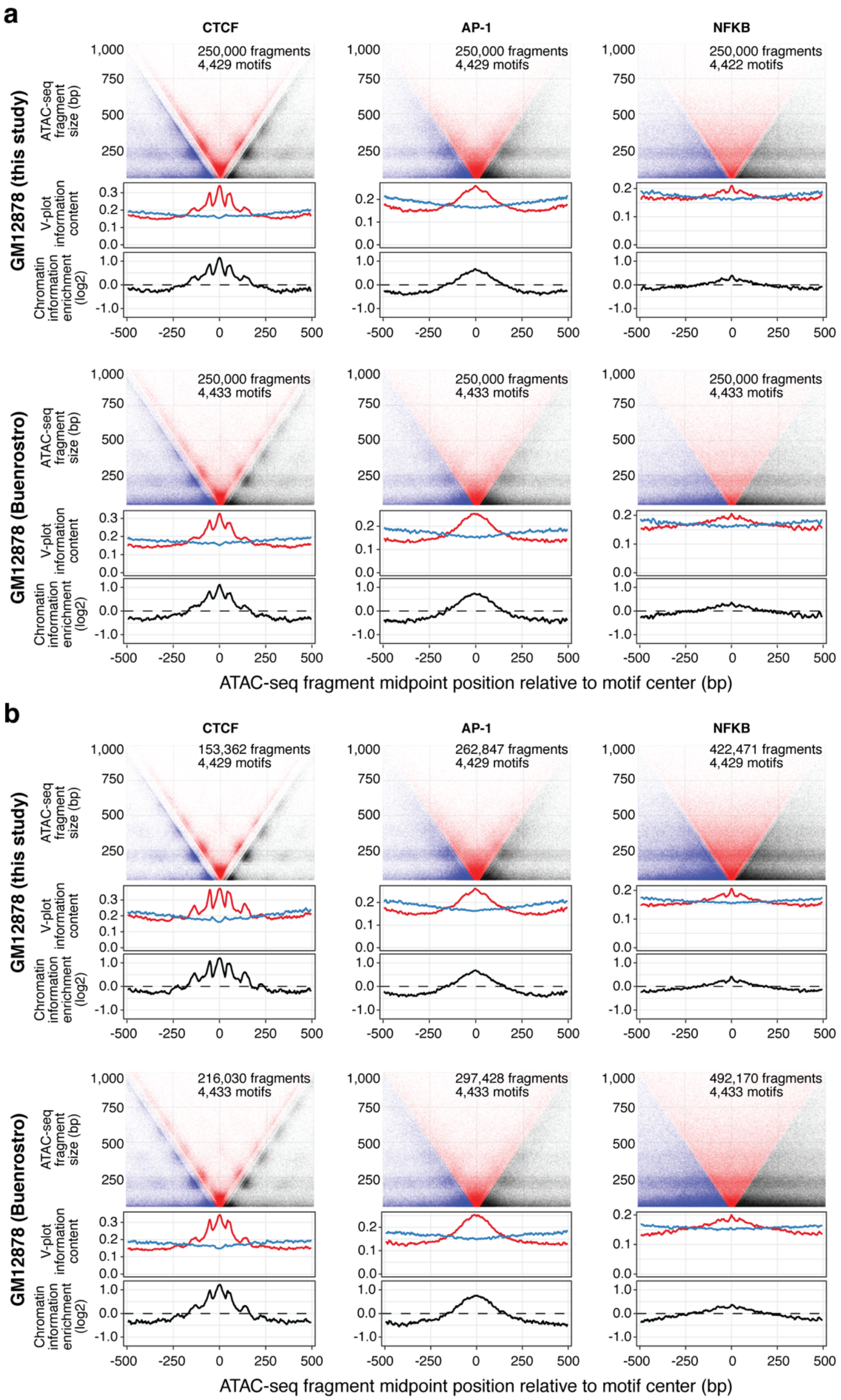
a
CTCF
AP-1
NFKB
GM12878 (this study)
ATAC-seq fragment size (bp)
V-plot information content
Chromatin information enrichment (log2)
250,000 fragments
4,429 motifs
250,000 fragments
4,429 motifs
250,000 fragments
4,422 motifs
GM12878 (Buenrostro)
250,000 fragments
4,433 motifs
250,000 fragments
4,433 motifs
250,000 fragments
4,433 motifs
ATAC-seq fragment midpoint position relative to motif center (bp)
b
CTCF
AP-1
NFKB
GM12878 (this study)
153,362 fragments
4,429 motifs
262,847 fragments
4,429 motifs
422,471 fragments
4,429 motifs
GM12878 (Buenrostro)
216,030 fragments
4,433 motifs
297,428 fragments
4,433 motifs
492,170 fragments
4,433 motifs
ATAC-seq fragment midpoint position relative to motif center (bp)

Supplementary Figure 3. **GM12878 V-plots.**
a V-plots for the same TFs as in Figure 1b across GM12878 datasets. Upper: ATAC-seq fragment distribution. Middle: observed (red) and expected (blue) information content tracks, used to calculate chromatin information enrichments (bottom). V-plots were downsampled to equal number of ATAC-seq fragments and motifs between TFs by selecting the top *n* motifs, ranked by number of ATAC-seq fragments, and then further downsampling to 250,000 fragments. *n* represents the smallest number of bound motifs among the plotted TFs per sample. **b** Similar to **a**, but randomly downsampling to exactly *n* motifs (without ranking by signal or further downsampling the number of ATAC-seq fragments). This was performed to demonstrate that the differences in chromatin architecture are intrinsic to the TF and evident regardless of downsampling method.

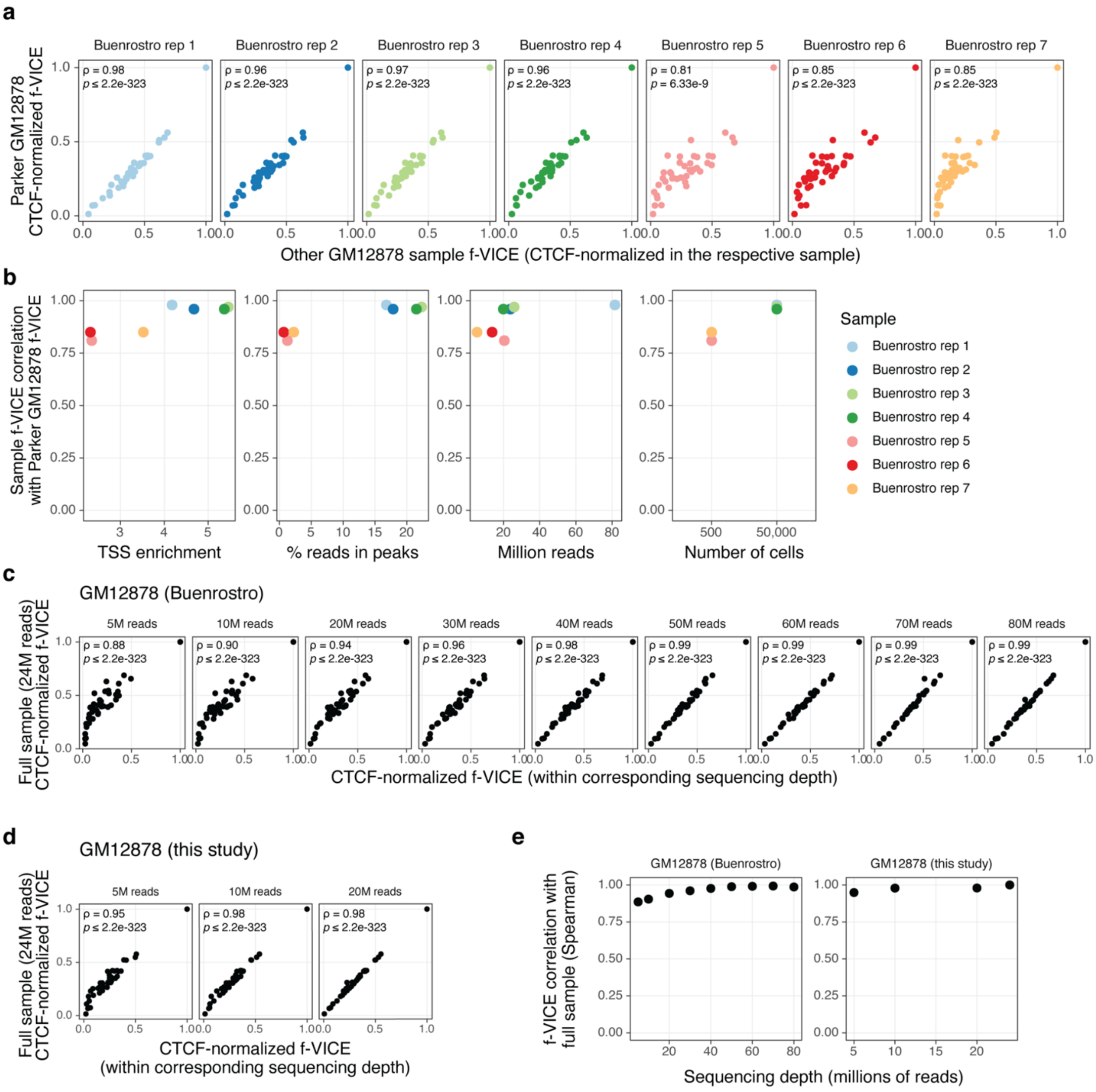

a
Buenrostro rep 1
Buenrostro rep 2
Buenrostro rep 3
Buenrostro rep 4
Buenrostro rep 5
Buenrostro rep 6
Buenrostro rep 7
ρ = 0.98 p ≤ 2.2e-323
ρ = 0.96 p ≤ 2.2e-323
ρ = 0.97 p ≤ 2.2e-323
ρ = 0.96 p ≤ 2.2e-323
ρ = 0.81 p = 6.33e-9
ρ = 0.85 p ≤ 2.2e-323
ρ = 0.85 p ≤ 2.2e-323
Parker GM12878 CTCF-normalized f-VICE
Other GM12878 sample f-VICE (CTCF-normalized in the respective sample)
b
Sample f-VICE correlation with Parker GM12878 f-VICE
TSS enrichment
% reads in peaks
Million reads
Number of cells
Sample
c
GM12878 (Buenrostro)
5M reads
10M reads
20M reads
30M reads
40M reads
50M reads
60M reads
70M reads
80M reads
ρ = 0.88 p ≤ 2.2e-323
ρ = 0.90 p ≤ 2.2e-323
ρ = 0.94 p ≤ 2.2e-323
ρ = 0.96 p ≤ 2.2e-323
ρ = 0.98 p ≤ 2.2e-323
ρ = 0.99 p ≤ 2.2e-323
ρ = 0.99 p ≤ 2.2e-323
ρ = 0.99 p ≤ 2.2e-323
ρ = 0.99 p ≤ 2.2e-323
Full sample (24M reads) CTCF-normalized f-VICE
CTCF-normalized f-VICE (within corresponding sequencing depth)
d
GM12878 (this study)
5M reads
10M reads
20M reads
ρ = 0.95 p ≤ 2.2e-323
ρ = 0.98 p ≤ 2.2e-323
ρ = 0.98 p ≤ 2.2e-323
Full sample (24M reads) CTCF-normalized f-VICE
CTCF-normalized f-VICE (within corresponding sequencing depth)
e
GM12878 (Buenrostro)
GM12878 (this study)
f-VICE correlation with full sample (Spearman)
Sequencing depth (millions of reads)

Supplementary Figure 4. Robustness of f-VICEs to noise metrics.
a Scatter plots of f-VICEs calculated from different Buenrostro GM12878 ATAC-seq libraries, which have varied signal-to-noise ratios (TSS enrichment between 2.2 and 5.5, fraction of read in peaks between 1% and 44%). Each point corresponds to a TF with ChIP-seq data in GM12878. f-VICE values for each sample were normalized using the corresponding CTCF f-VICE value from that sample. **b** Spearman correlation values from **a**. **c** Scatter plots of CTCF-normalized f-VICEs across different sequencing depths in the Buenrostro GM12878 ATAC-seq data compared to the f-VICEs calculated using the full sample. **d** Similar to **c**, but for the GM12878 ATAC-seq data generated for this study. Each point corresponds to a TF with ChIP-seq data. The same set of binding sites per TF were used to calculate f-VICEs. f-VICE values for each sequencing depth were normalized using the corresponding CTCF f-VICE value obtained from that sequencing depth. **e** Spearman correlation values for the facets displayed in **c** and **d**.

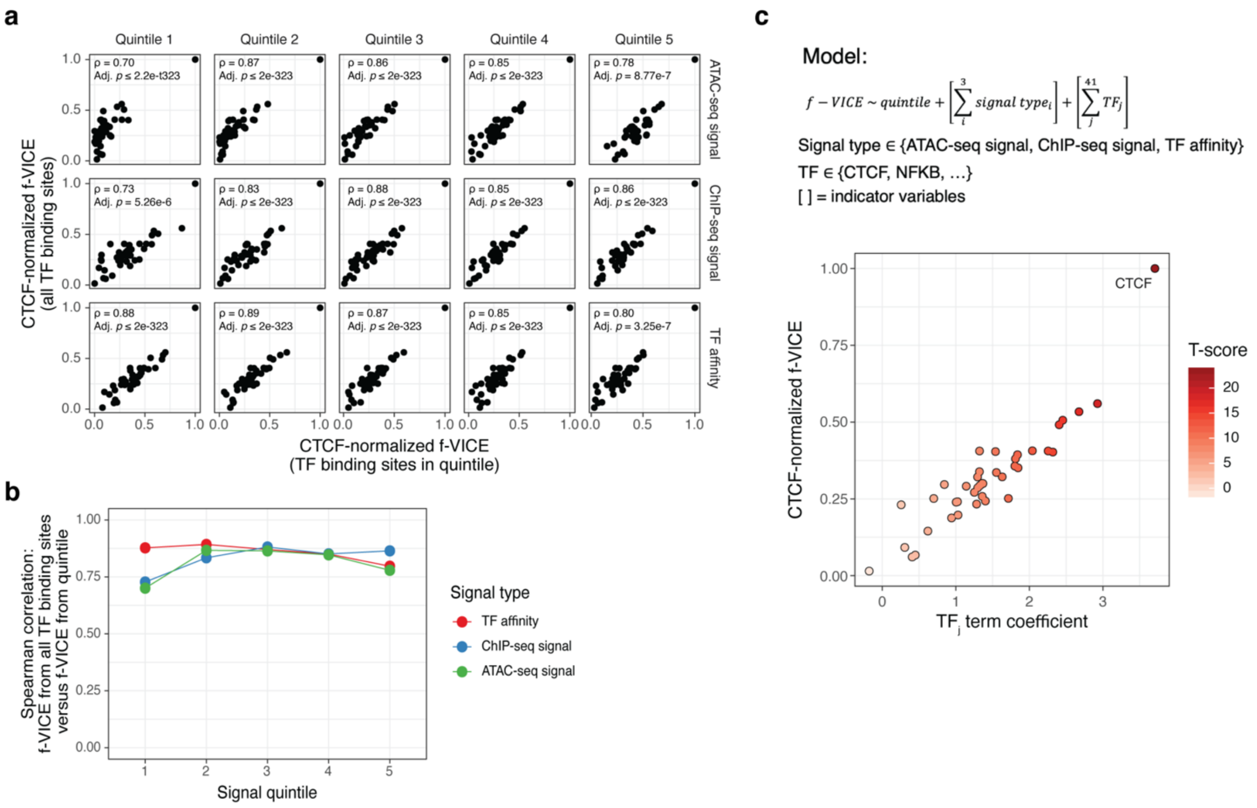

Supplementary Figure 5. TF f-VICEs are independent of other signal metrics.
a Scatter plots of GM12878 CTCF-normalized f-VICEs calculated using only the TF binding site instances corresponding to each signal type quintile (indicated in the rows and column facets) versus the f-VICEs calculated from all TF-bound motif instances. Each point corresponds to a TF with ChIP-seq data. All f-VICE values on the x-axis are normalized by the CTCF value of the subset of the data indicated in the facets (metric/quintile). The f-VICE values on the y-axis are normalized by the CTCF value of the full dataset. **b** Spearman correlations for all comparisons. Each point corresponds to one of the facets in **a.** **c** Scatter plot of the TF term coefficients from the linear model (described in the top of the panel) versus f-VICEs calculated using all bound motif instances. Each point corresponds to a TF with ChIP-seq data.

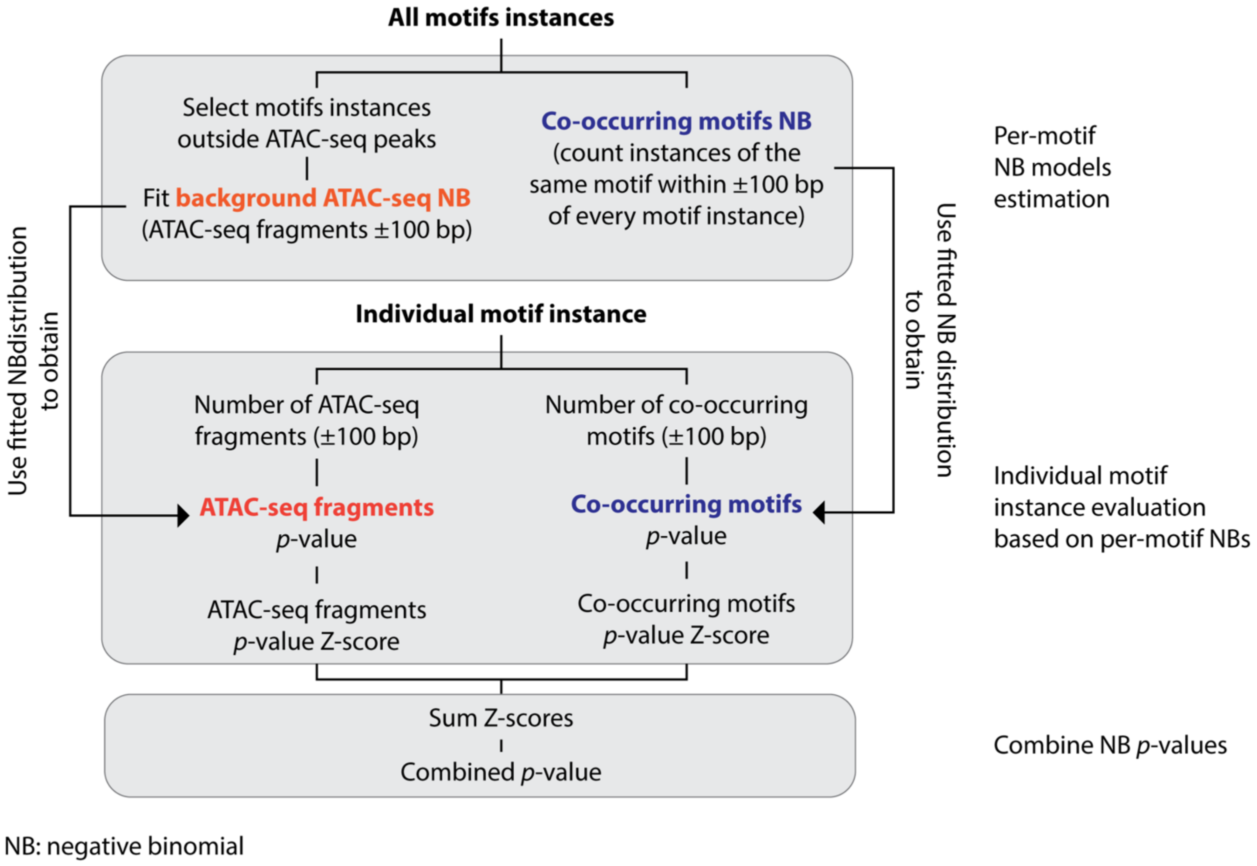

Supplementary Figure 6. BMO schematic.
Schematic overview of the approach used to predict TF binding using BMO.

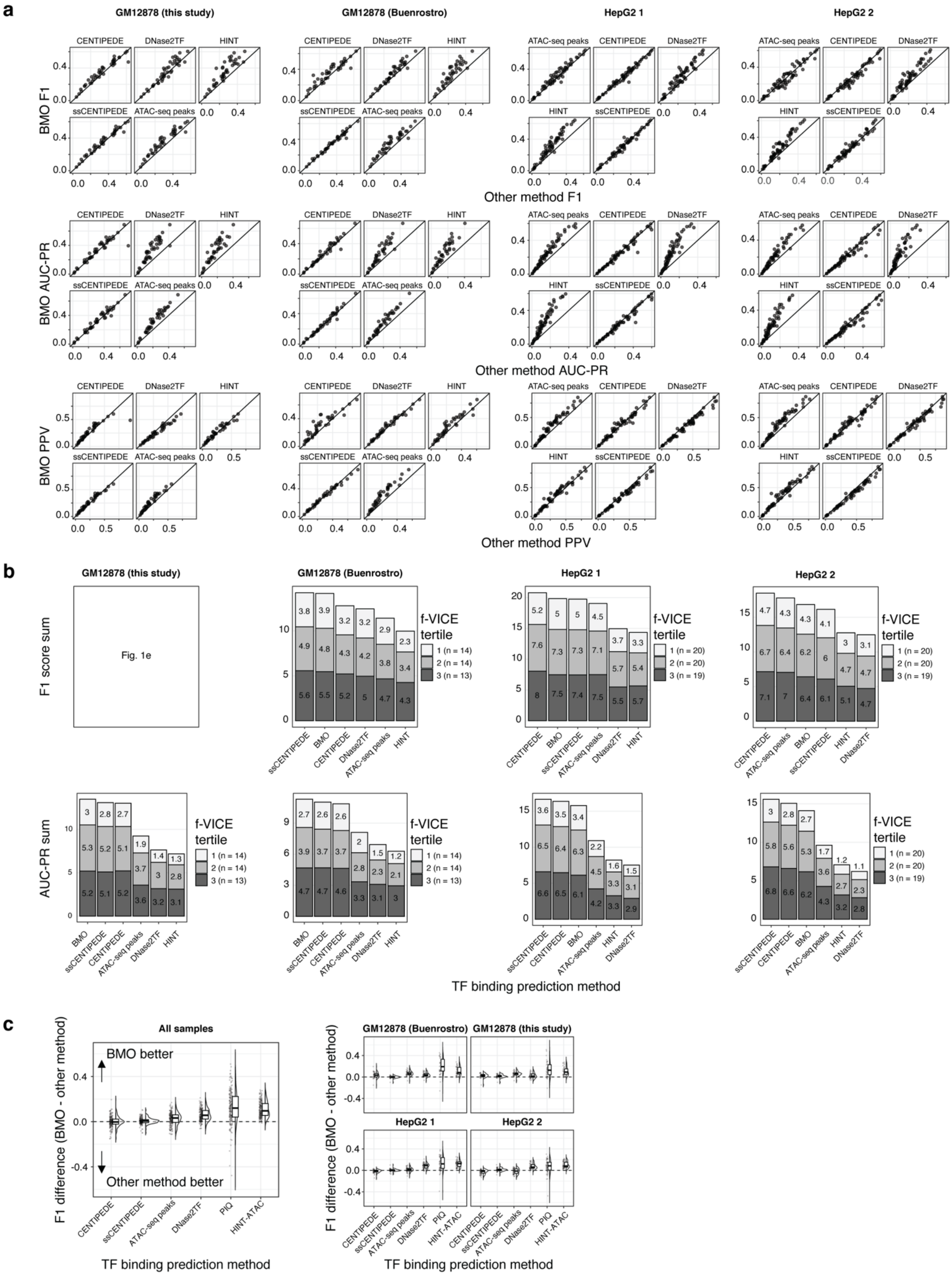

a
GM12878 (this study)
GM12878 (Buenrostro)
HepG2 1
HepG2 2
BMO F1
Other method F1
BMO AUC-PR
Other method AUC-PR
BMO PPV
Other method PPV
CENTIPEDE
DNase2TF
HINT
ssCENTIPEDE
ATAC-seq peaks
b
F1 score sum
Fig. 1e
AUC-PR sum
f-VICE tertile
1 (n = 14)
2 (n = 14)
3 (n = 13)
1 (n = 20)
2 (n = 20)
3 (n = 19)
TF binding prediction method
c
All samples
BMO better
Other method better
F1 difference (BMO - other method)
PIQ
HINT-ATAC
TF binding prediction method

Supplementary Figure 7. TF binding prediction methods comparisons across datasets.

a F1, positive predictive value (PPV), and AUC-PR scatter plots of BMO versus other TF binding prediction methods across multiple ATAC-seq datasets. Each point corresponds to a TF with ChIP-seq data. Solid diagonal line, identity ($x = y$). **b** Total F1-score and AUC-PR across datasets, separated into f-VICE tertiles. **c** F1 difference between BMO and the other methods for all comparisons (left) and for each sample (right). Each point corresponds to a TF with ChIP-seq data (n = 41 and n = 59 for GM12878 and HepG2, respectively). Boxplots centers, boxes, and whiskers represent median, 1st and 3rd quartiles, and 1.5 interquartile range, respectively.

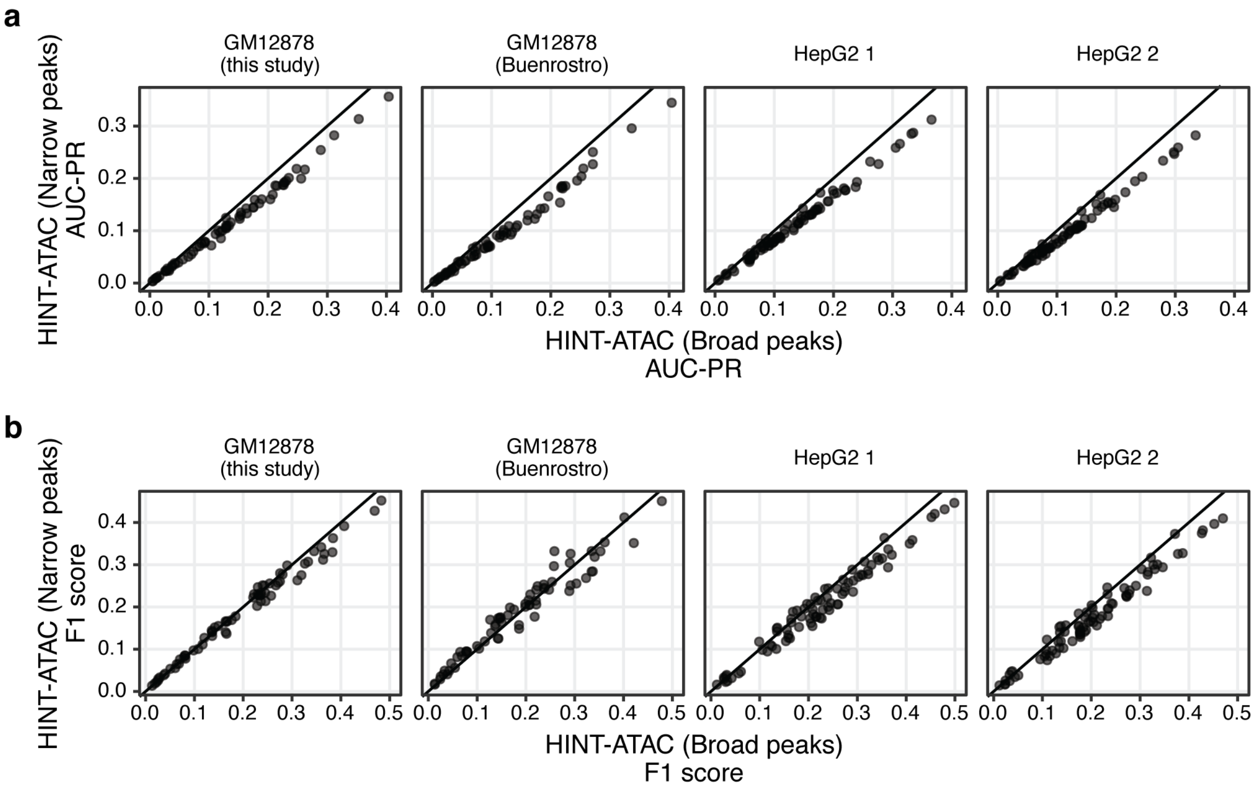

Supplementary Figure 8. HINT-ATAC performance using narrow or broad peak calls. Scatter plots of AUC-PRs (**a**) and F1 scores (**b**) across datasets. Each point corresponds to a TF with ChIP-seq data (*n* = 41 and *n* = 59 for GM12878 and HepG2, respectively). Solid diagonal line, identity (*x* = *y*).

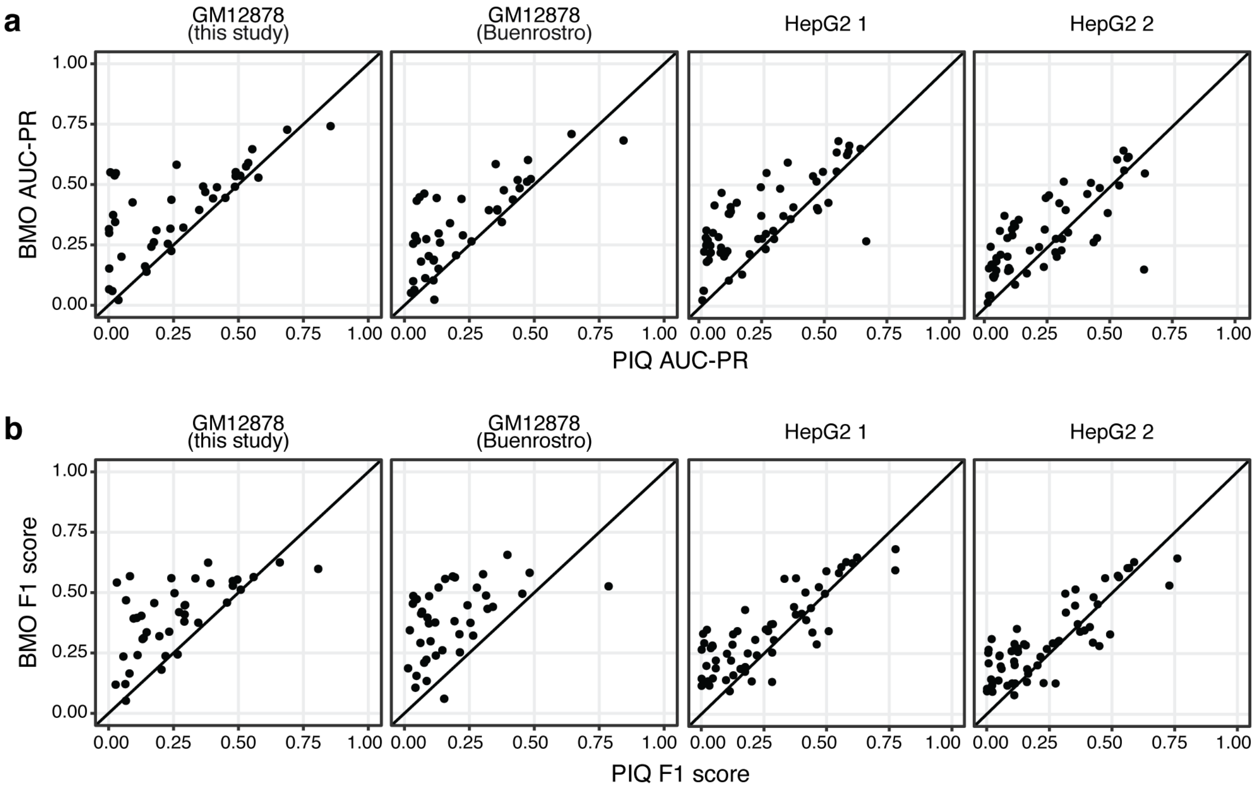

Supplementary Figure 9. **BMO and PIQ comparisons.**
Scatter plots of AUC-PR (**a**) and F1 scores (**b**) across datasets comparing BMO and PIQ. Each point corresponds to a TF with ChIP-seq data (n = 41 and n = 59 for GM12878 and HepG2, respectively). Solid diagonal line, identity ($x = y$).

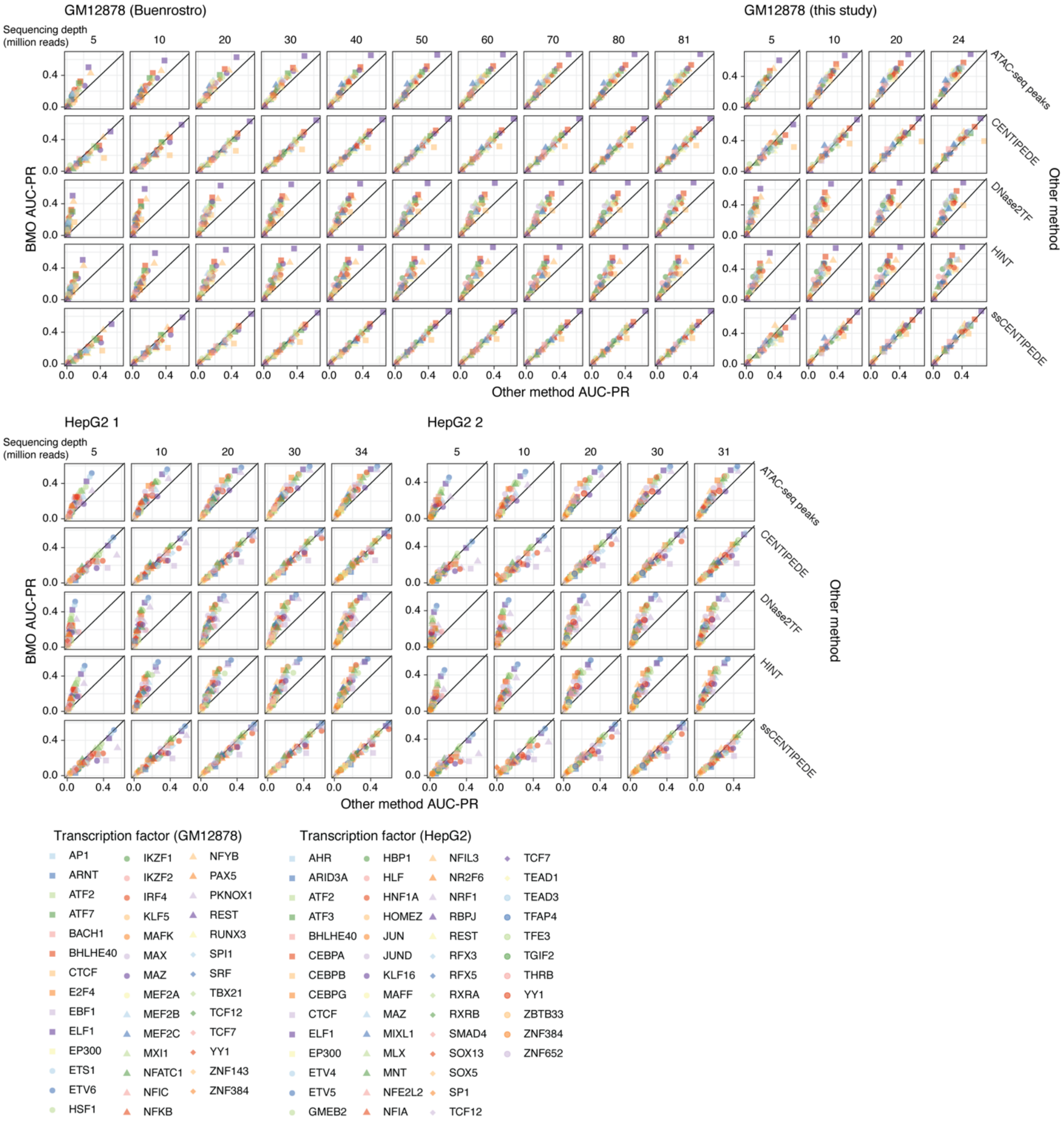

Supplementary Figure 10. TF binding prediction methods comparisons across sequencing depths. AUC-PR scatter plots of BMO versus other methods across different sequencing depths, shown in millions of reads in the top of each facet column. Each point corresponds to one TF with ChIP-seq data data (n = 41 and n = 59 for GM12878 and HepG2, respectively). Solid diagonal lines, identity ($x = y$).

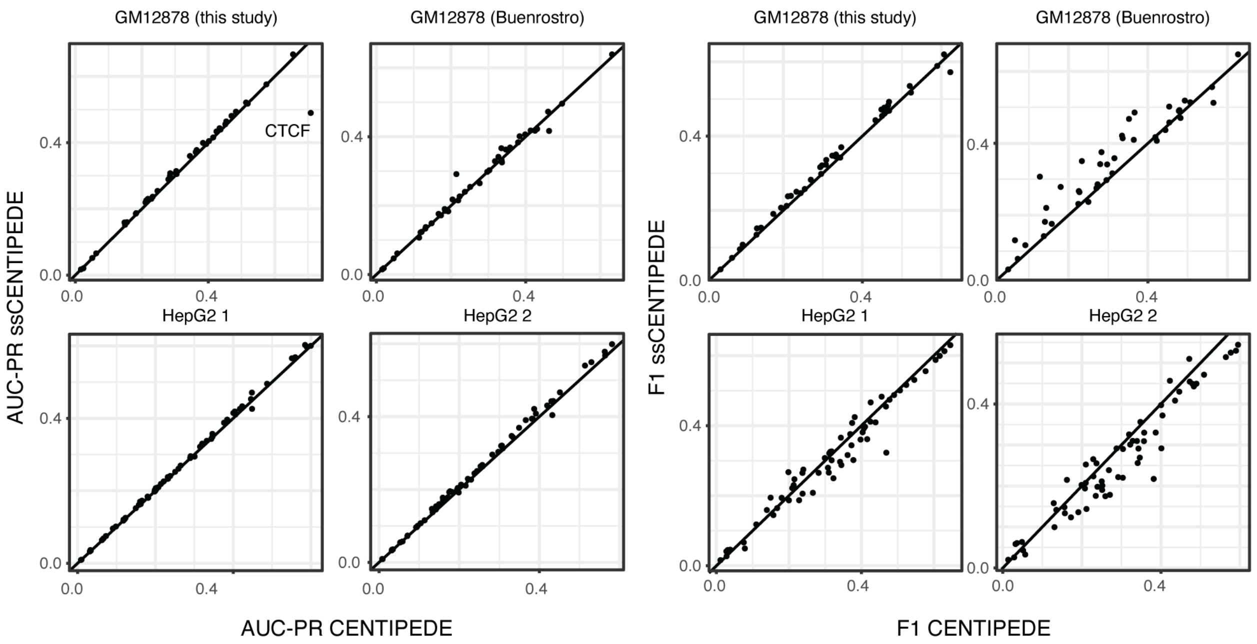

Supplementary Figure 11. CENTIPEDE and ssCENTIPEDE perform similarly across datasets.

Scatter plots of ssCENTIPEDE and CENTIPEDE AUC-PRs and F1 scores across multiple datasets. Each point corresponds to one TF with ChIP-seq data data (n = 41 and n = 59 for GM12878 and HepG2, respectively). Solid diagonal line, identity ($x = y$).

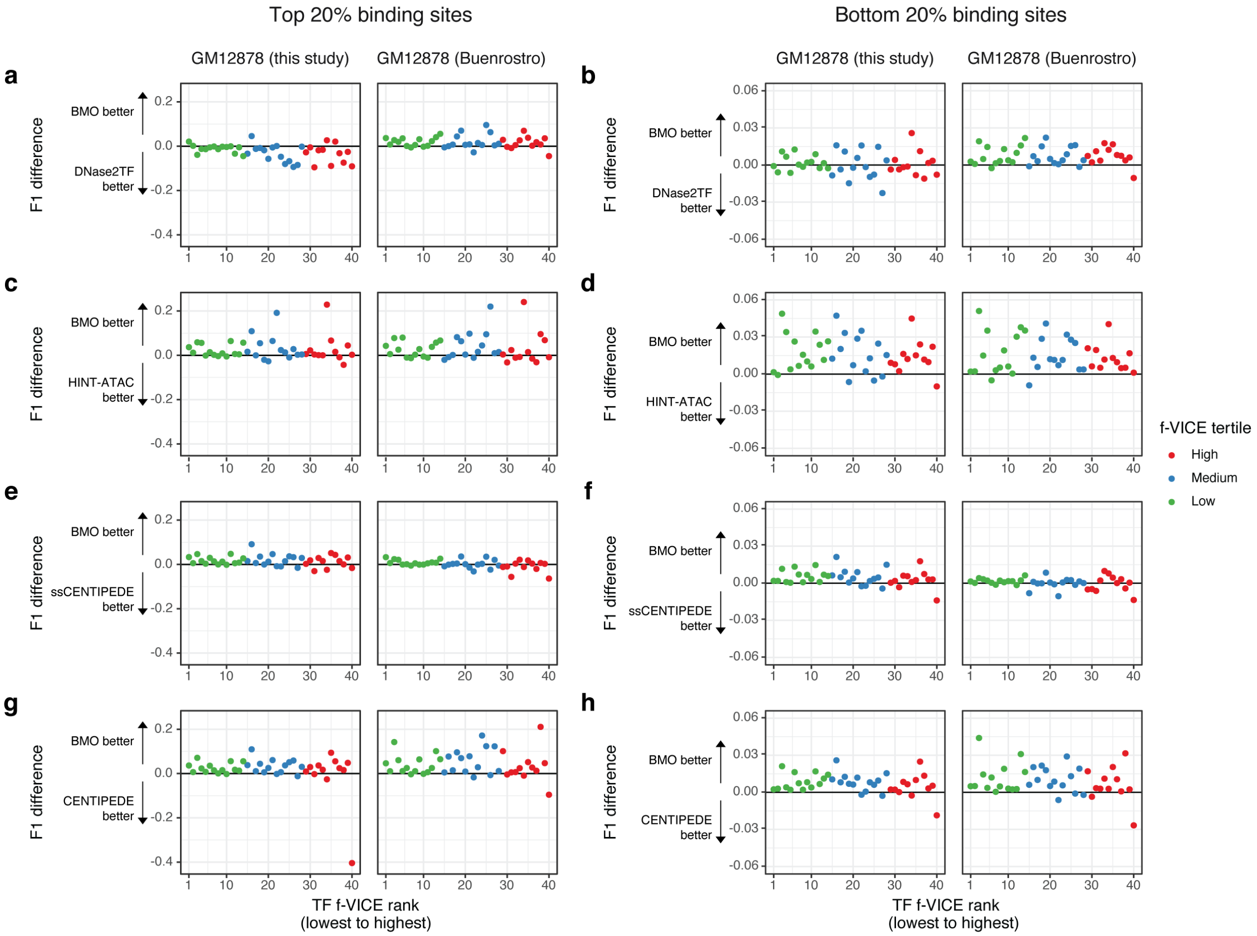

Supplementary Figure 12. Effect of TF binding affinity on predictions.
TF binding predictions of the TF binding sites intersecting the top (**a**, **c**, **e**, and **g**) and bottom (**b**, **d**, **f**, and **h**) 20% ChIP-seq peaks. Each point corresponds to a TF with ChIP-seq data in GM12878 data (n = 41). The x-axis corresponds to the f-VICE rank of the TF (from lowest to highest f-VICE), calculated using the high-occupancy motif instances. The y-axes correspond to the difference in F1 scores between BMO and the other methods for that TF. Values greater than zero indicate that BMO outperformed the other method when predicting that TF.

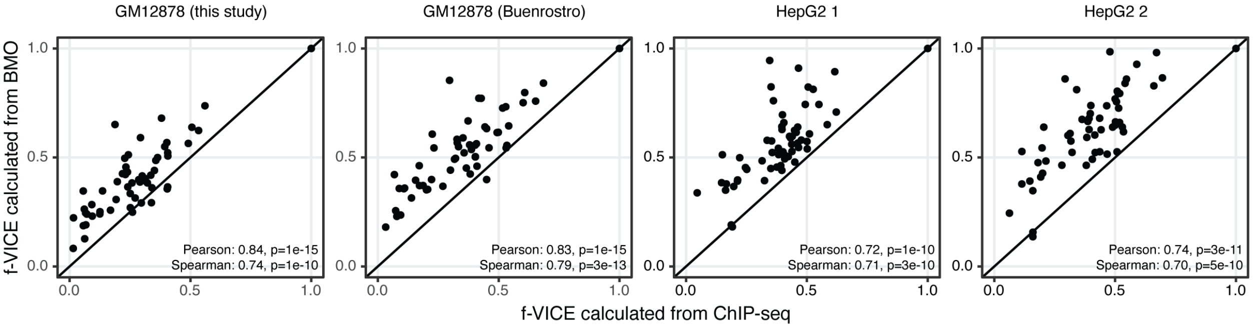

Supplementary Figure 13. BMO and ChIP-seq f-VICEs are correlated.
Correlation of f-VICEs calculated from BMO predictions and from the respective ChIP-seq data across ATAC-seq datasets data (*n* = 41 and *n* = 59 for GM12878 and HepG2, respectively). Note that BMO f-VICEs are consistently higher than ChIP-seq f-VICEs. This is due to a higher number of predicted bound motif instances in BMO, which motivated us to normalize f-VICEs using the linear regression approach described in the methods (f-VICEs are not normalized using regression in this figure owing to low *n*). Solid diagonal line, identity (*x* = *y*).

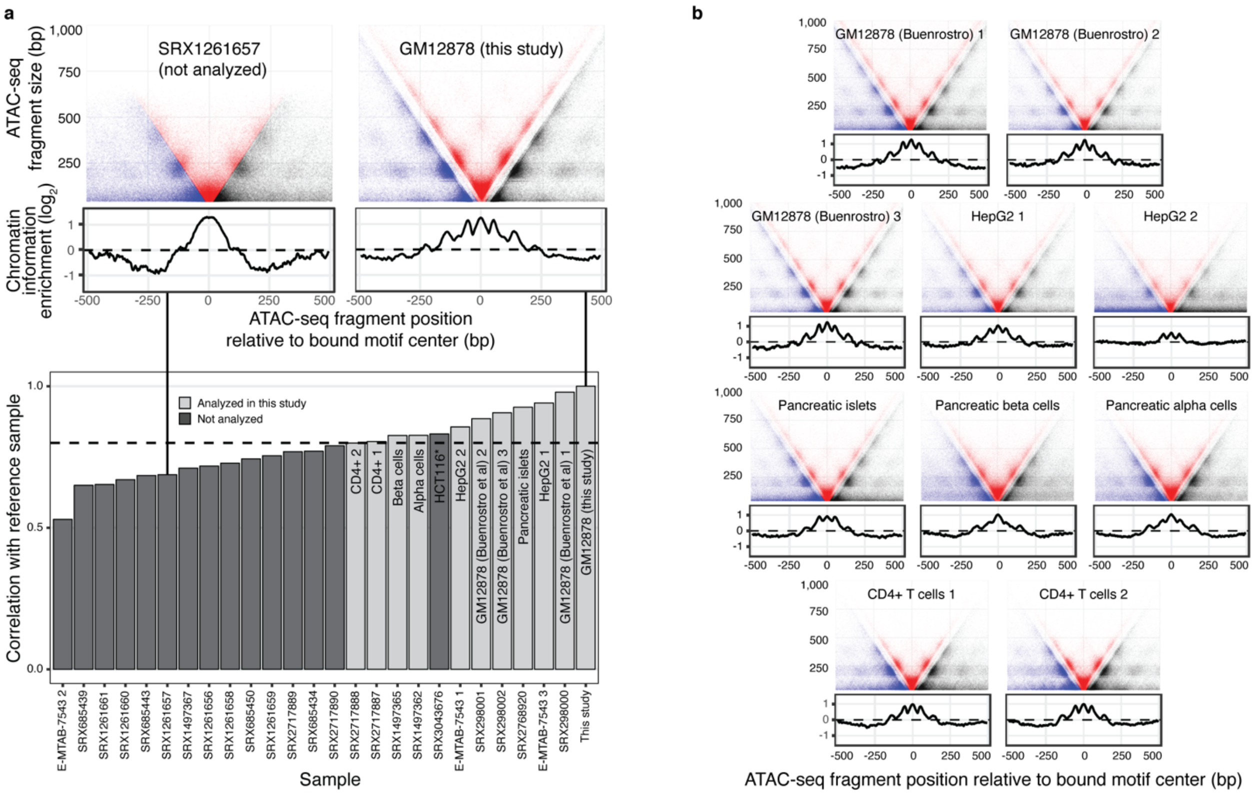

* Did not have another sample of the same tissue/cell line that passed QC

Supplementary Figure 14. Selection of additional ATAC-seq samples using ubiquitous and conserved CTCF-cohesin binding sites.
a Upper: examples of V-plots for the reference ubiquitous and conserved CTCF-cohesin binding sites indicating a high-quality and low-quality sample (the latter shown for exemplification purposes and not included in this study). Lower: chromatin information enrichment correlation between the CTCF-cohesin binding sites across multiple experiments to a reference sample. **b** V-plots of the same regions in the other samples selected for this study. Y-axes labels are the same as the upper plot in **a**.

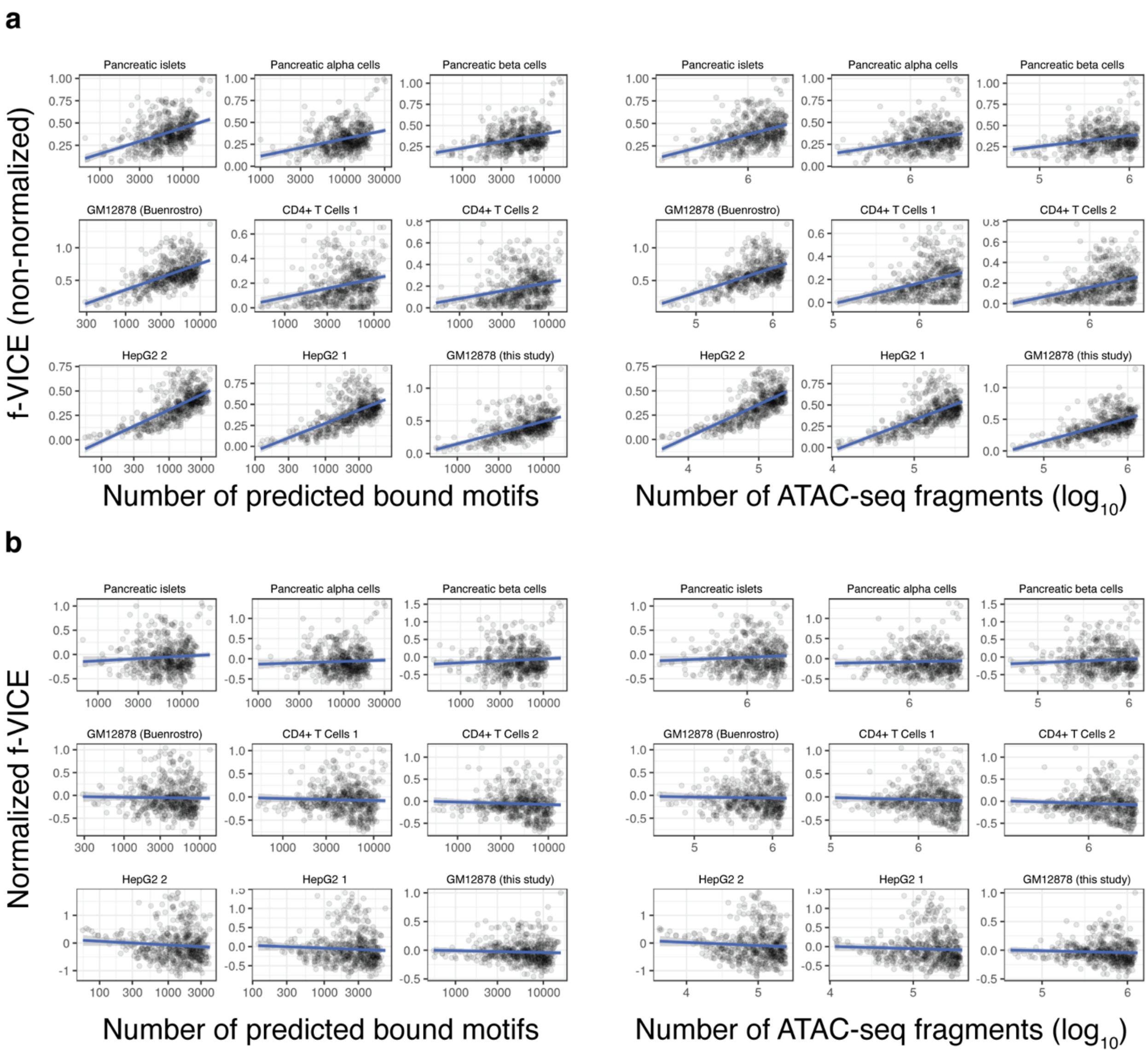

Supplementary Figure 15. Normalization of f-VICE.

a Scatter plots of f-VICE as a function of number of predicted bound motifs or ATAC-seq signal. **b** Same data after normalization using a linear regression model that accounts for both variables (described in the Methods section). Each point corresponds to a motif (n = 540).

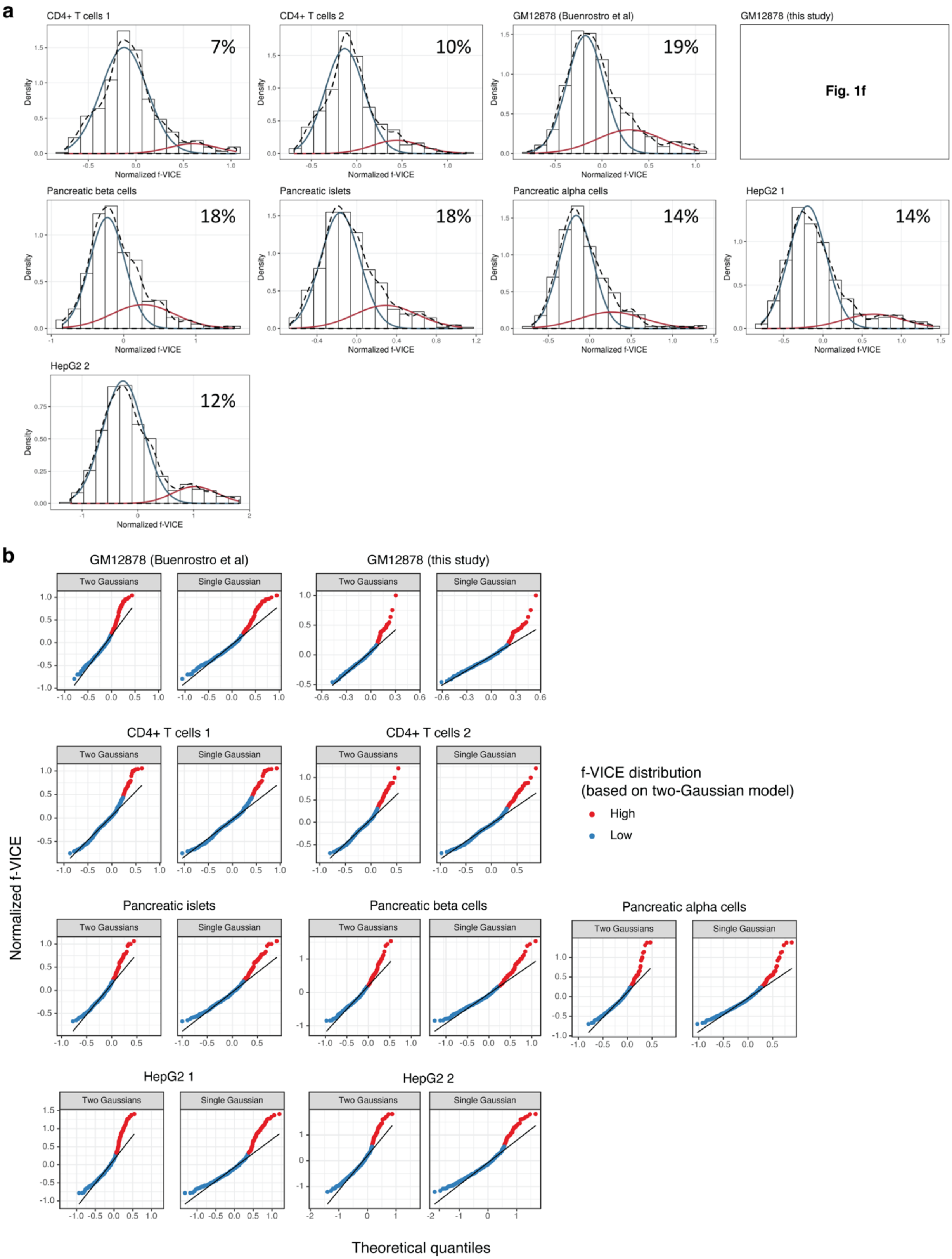
a
CD4+ T cells 1
7%
CD4+ T cells 2
10%
GM12878 (Buenrostro et al)
19%
GM12878 (this study)
Fig. 1f
Pancreatic beta cells
18%
Pancreatic islets
18%
Pancreatic alpha cells
14%
HepG2 1
14%
HepG2 2
12%
Density
Normalized f-VICE
b
GM12878 (Buenrostro et al)
GM12878 (this study)
CD4+ T cells 1
CD4+ T cells 2
Pancreatic islets
Pancreatic beta cells
Pancreatic alpha cells
HepG2 1
HepG2 2
Two Gaussians
Single Gaussian
f-VICE distribution
(based on two-Gaussian model)
High
Low
Normalized f-VICE
Theoretical quantiles

Supplementary Figure 16. f-VICE distributions across samples.

a Histograms and density plots of the empirical (dashed) and high/low f-VICE distributions Gaussian fits (red and blue, respectively) across all the datasets surveyed in this work. Percentages in the upper right corner of plots represent the high f-VICE distribution. **b** Q-Q plots of the f-VICE values across all samples. The x-axis corresponds to the theoretical quantiles calculated using the parameters (mean and SD) of the low f-VICE Gaussian obtained from the mixture model (left) or the parameters obtained from fitting a single Gaussian to the data (right). The y-axis corresponds to the normalized f-VICE sample distribution. Solid black line, slope and intercept connecting the theoretical quantiles to the sample distribution. Colors represent the high (red) and low (blue) f-VICE distribution assignments from the two Gaussians model.

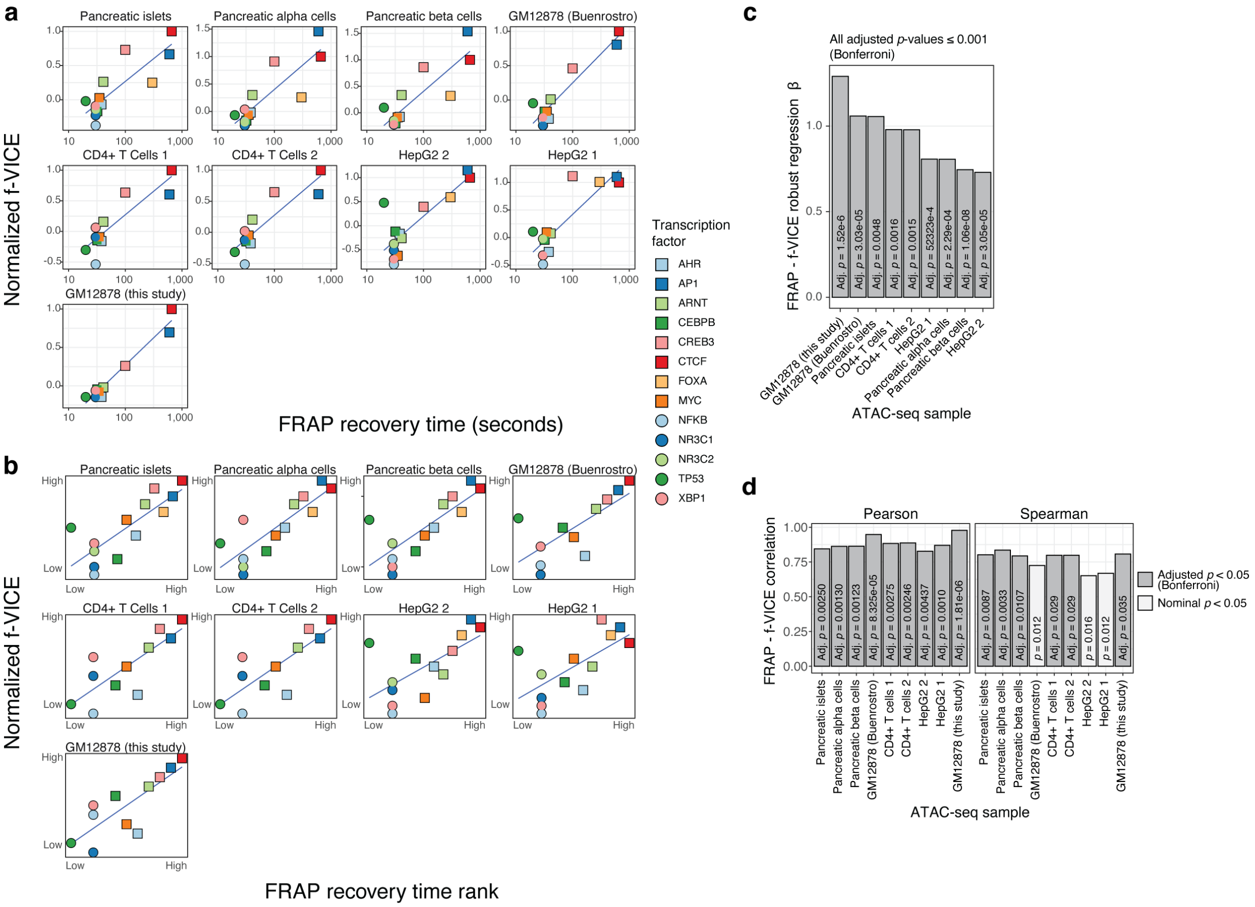

Supplementary Figure 17. f-VICE correlation with FRAP recovery times in multiple datasets.

a Scatter plots of mammalian FRAP recovery times and f-VICEs across multiple datasets. **b** Similar to **a**, but showing f-VICE and FRAP ranks (similar to a Spearman correlation). Solid blue lines, linear model fit. **c** Robust linear regression betas (model: f-VICE ~ FRAP recovery time) for the plots shown in **a**. Adjusted *p* values calculated using Bonferroni correction. **d** Pearson and Spearman correlations of the plots shown in **a**.

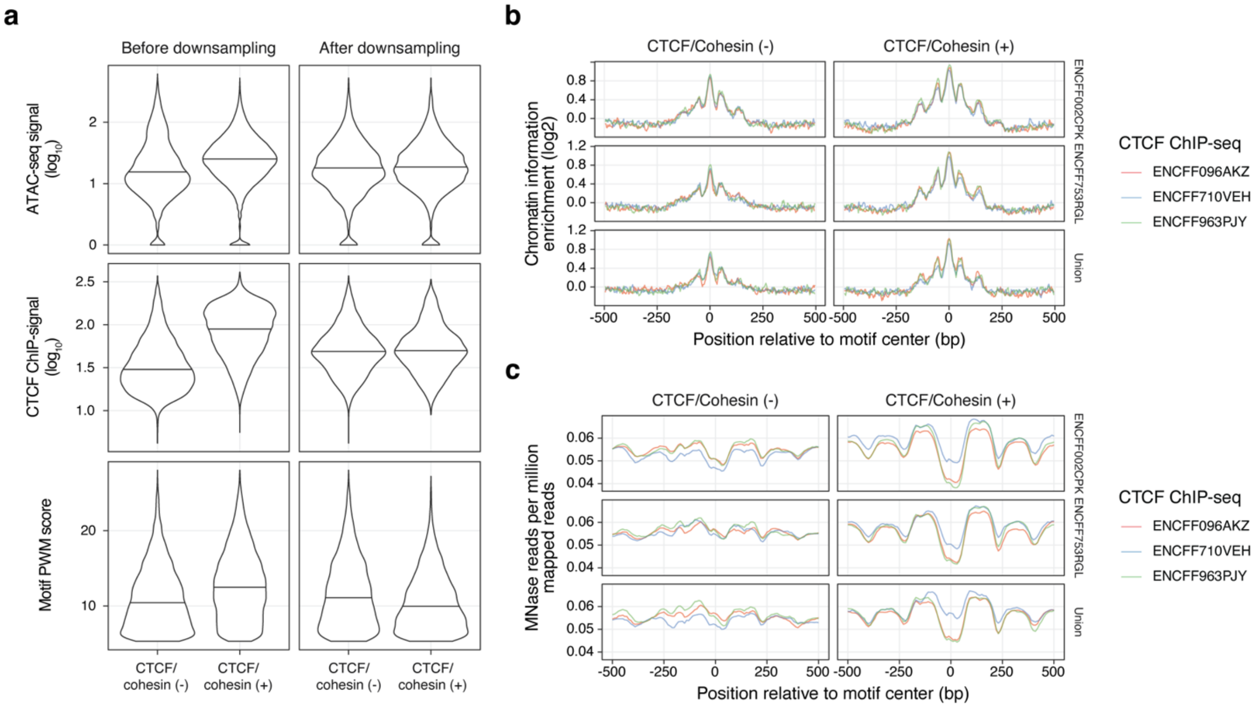

Supplementary Figure 18. GM12878 CTCF/cohesin^{+} and CTCF/cohesin^{-} regions.
a Example distributions of ATAC-seq signal, ChIP-seq signal, and motif PWM match score before and after quantile-based downsampling of the CTCF/cohesin^{+} and CTCF/cohesin^{-}. Datasets: ENCFF963PJY (CTCF) and ENCFF002CPK (Rad21). Other CTCF/Rad21 ChIP-seq dataset combinations not shown. Horizontal lines, median. **b** Chromatin information tracks of CTCF/cohesin^{+} and CTCF/cohesin^{-} using genomic regions obtained from different GM12878 CTCF and RAD21 ChIP-seq datasets combinations. The facet labeled “Union” corresponds to the union of the two GM12878 RAD21 ChIP-seq datasets. **c** Corresponding MNase signal at the regions shown in **b**.

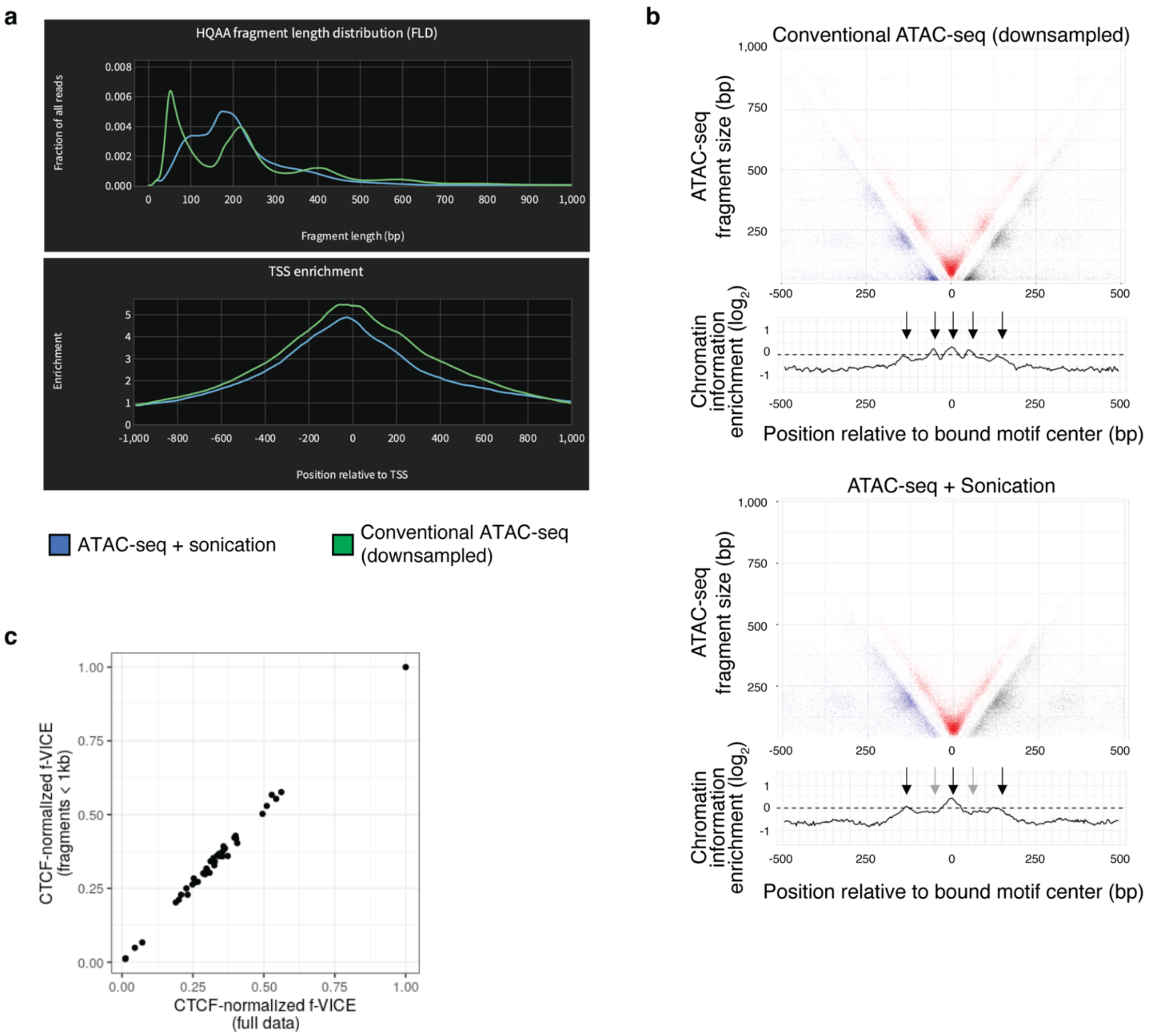

Supplementary Figure 19. Sonicated GM12878 ATAC-seq data.
a Ataqv[9] (github.com/ParkerLab/ataqv) screenshot showing the fragment size distribution and TSS enrichments of the conventional and sonicated GM12878 ATAC-seq datasets generated in this study. HQAA = high-quality autosomal alignments. **b** V-plots of the reference conserved CTCF-cohesin regions in the two datasets. "Conventional ATAC-seq" refers to the sample labeled as "GM12878 (this study)" in other figures. However, this dataset was downsampled to the same depth as the sonicated dataset (3.45 million reads) for the analyses presented in this figure and Figure 2a in order to make datasets directly comparable. Black arrows, CIE peaks in both samples. Gray arrows, CIE peaks not in the sonicated sample. **c** Effect of large ATAC-seq fragments on f-VICE calculations. Scatter plot between f-VICEs calculated using all ATAC-seq fragments versus f-VICE using fragments < 1 kb. Each data point corresponds to a motif with ChIP-seq data in GM12878 data (n = 41). ATAC-seq data: GM12878, this study.

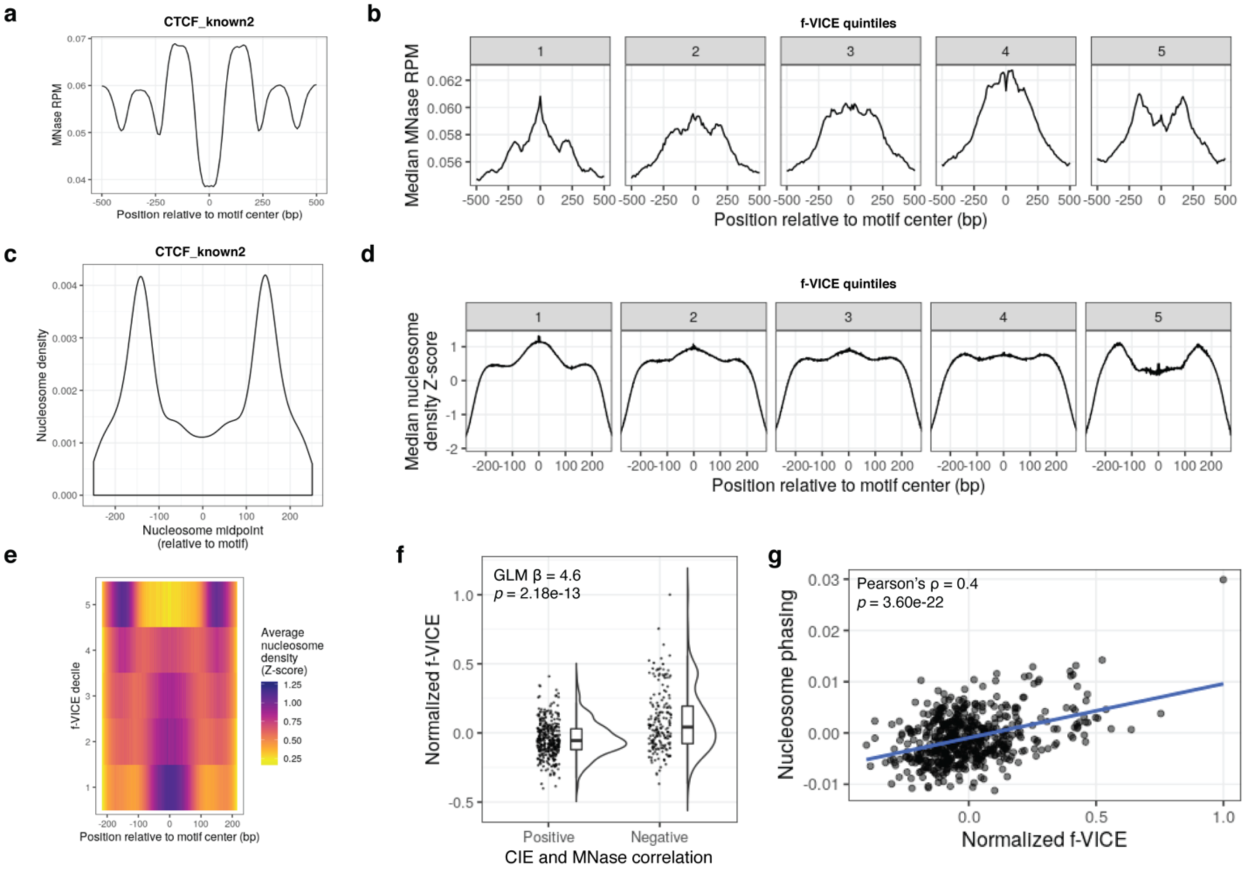

Supplementary Figure 20. Correlation between CIE and nucleosome occupancy patterns in GM12878.

a Example MNase-seq plot of CTCF_known2 predicted bound motifs. **b** Median MNase-seq reads per million (RPM) at predicted bound motifs across f-VICE quintiles. **c** Example plot showing the nucleosome midpoint density around predicted bound CTCF motifs estimated from the GM12878 data generated in this study using NucleoATAC[10]. **d** Median nucleosome occupancy patterns at predicted bound motifs across f-VICE quintiles estimated using NucleoATAC. Individual motif nucleosome densities were converted to Z-scores before calculating the median density per position. **e** Average nucleosome occupancy across motifs from different f-VICE quintiles, calculated using NucleoATAC. **f** f-VICE values for motifs with positive and negative CIE and MNase correlation (≤150 bp from motif center). Significance was calculated using a binomial generalized linear model (GLM) of f-VICE ~ CIE/MNase correlation (n = 540). Boxplots centers, boxes, and whiskers represent median, 1st and 3rd quartiles, and 1.5 interquartile range, respectively. **g** Correlation between f-VICE and the difference of the MNase signal at the motif vicinity (±125-150 bp from motif center) and the MNase signal at the motif (±25 bp from motif center). Positives ratios indicate nucleosome phasing. n = 540.

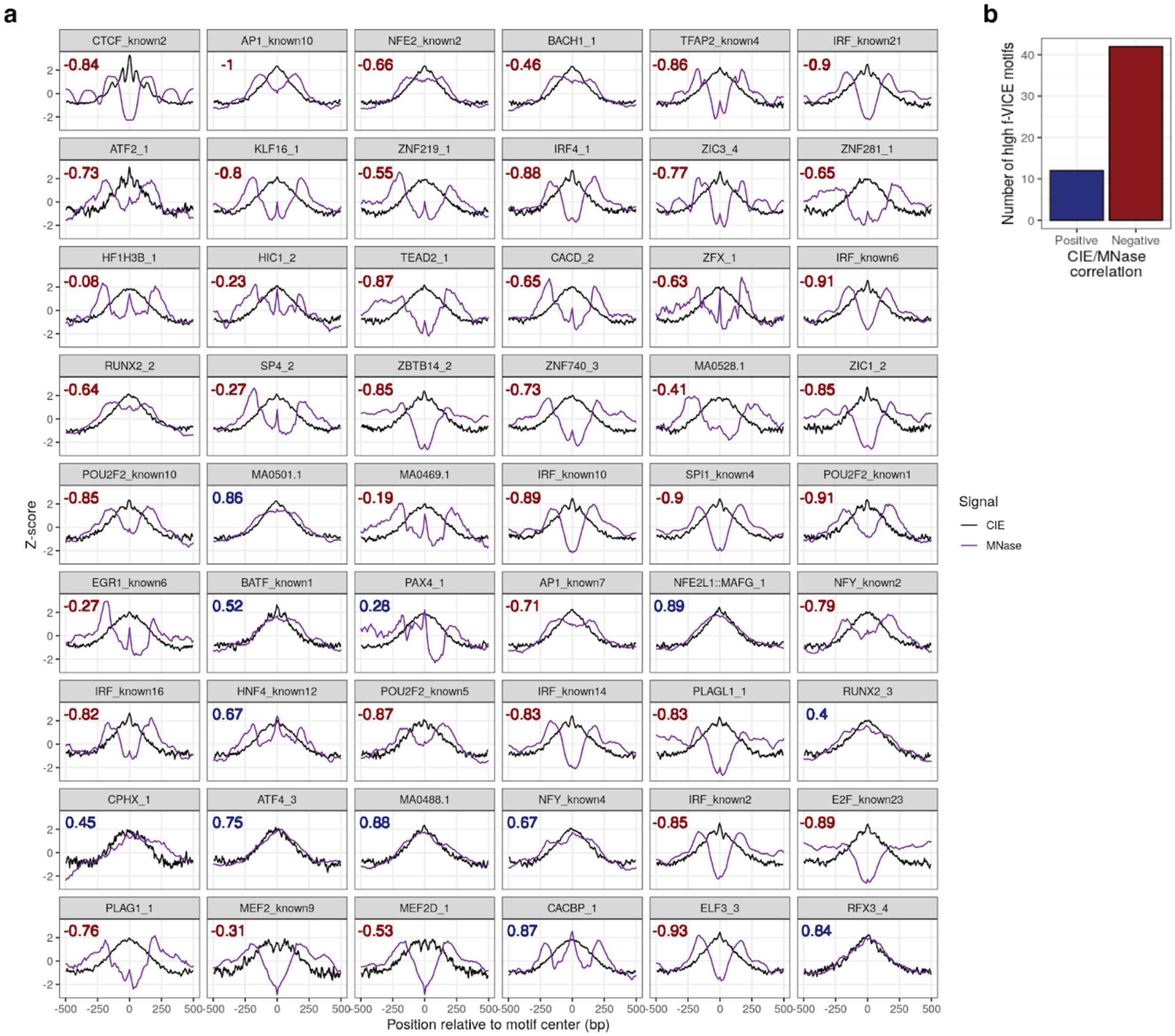

Supplementary Figure 21. High f-VICE motifs and their nucleosome occupancy patterns in GM12878.

a CIE (black) and MNase (purple) signals at high f-VICE motifs. Each facet in the plot represents one of the high f-VICE motifs based on the CIE distribution calculated in the GM12878 ATAC-seq data generated in this study. Values in the upper left correspond to the positive (dark blue) and negative (dark red) Spearman correlation values between CIE and MNase patterns (≤ 150 bp from motif center). **b** Number of high f-VICE motifs with either positive or negative CIE/MNase correlation.

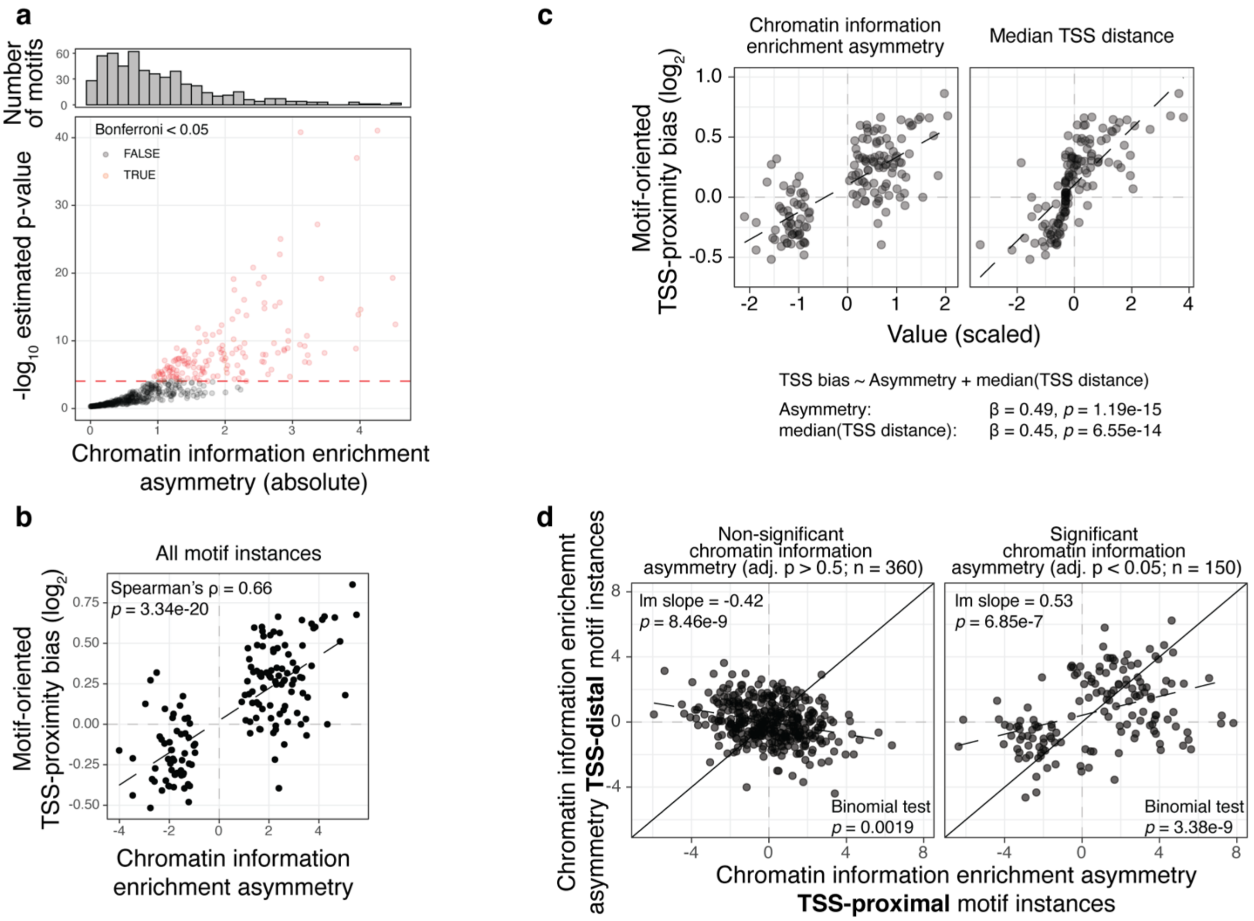

Supplementary Figure 22. **Motifs with information asymmetry in GM12878.**
a Chromatin information asymmetry distribution in GM12878. Red dashed line represent the Bonferroni *p*-value cutoff threshold. **b** Relationship between chromatin information asymmetry and motif-oriented TSS proximity bias based on all motif instances. **c** Motif-oriented TSS proximity bias chromatin as a function of information asymmetry and median nearest TSS distance. We performed a regression analysis of nearest TSS direction bias and chromatin information enrichment asymmetry, controlling for TSS distance (Methods). Chromatin information enrichment asymmetry remained significant when controlling for TSS distance. **d** Concordance of chromatin information asymmetry direction between TSS-distal and TSS-proximal motif instances. Solid diagonal line, identity ($x = y$). Dashed black lines, linear model (lm) fit in the data.

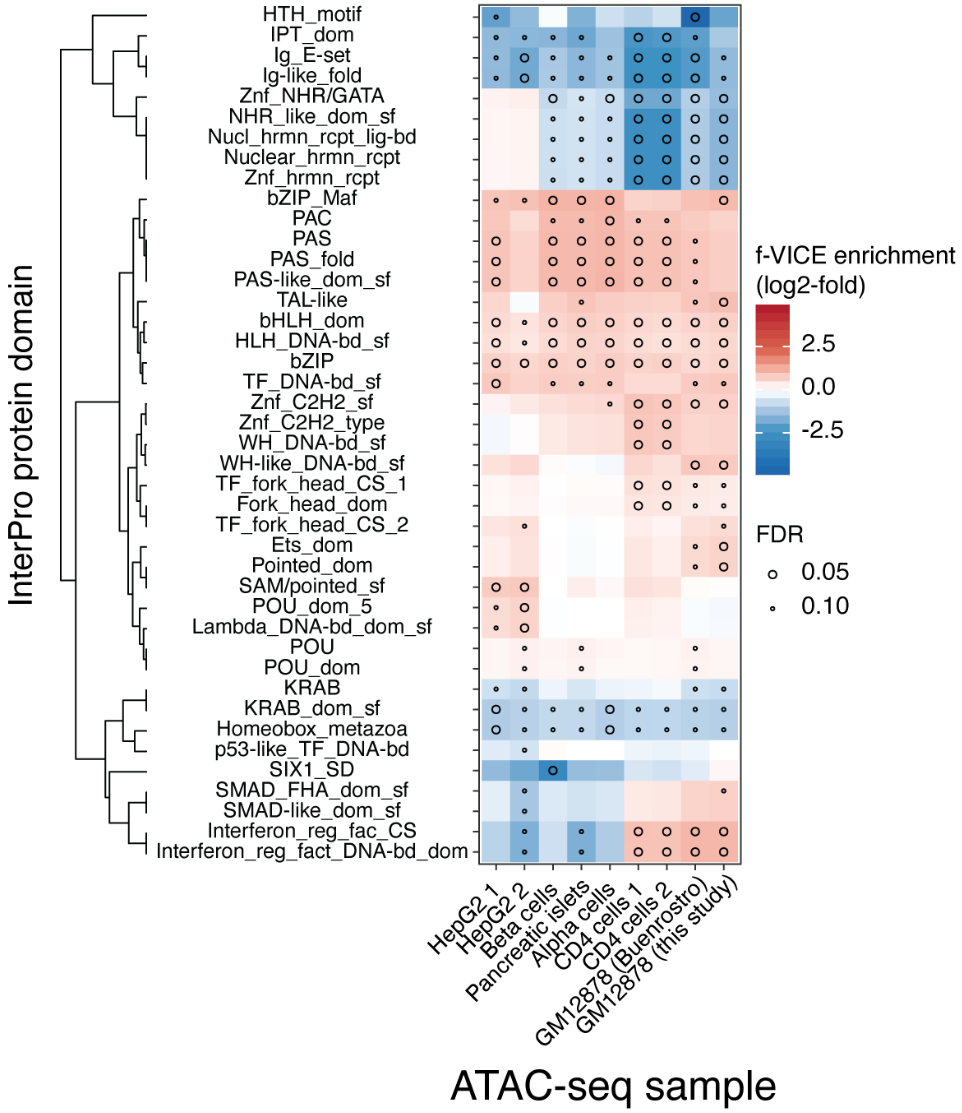

Supplementary Figure 23. Protein domain enrichments.
InterPro protein domains f-VICE enrichments across samples.

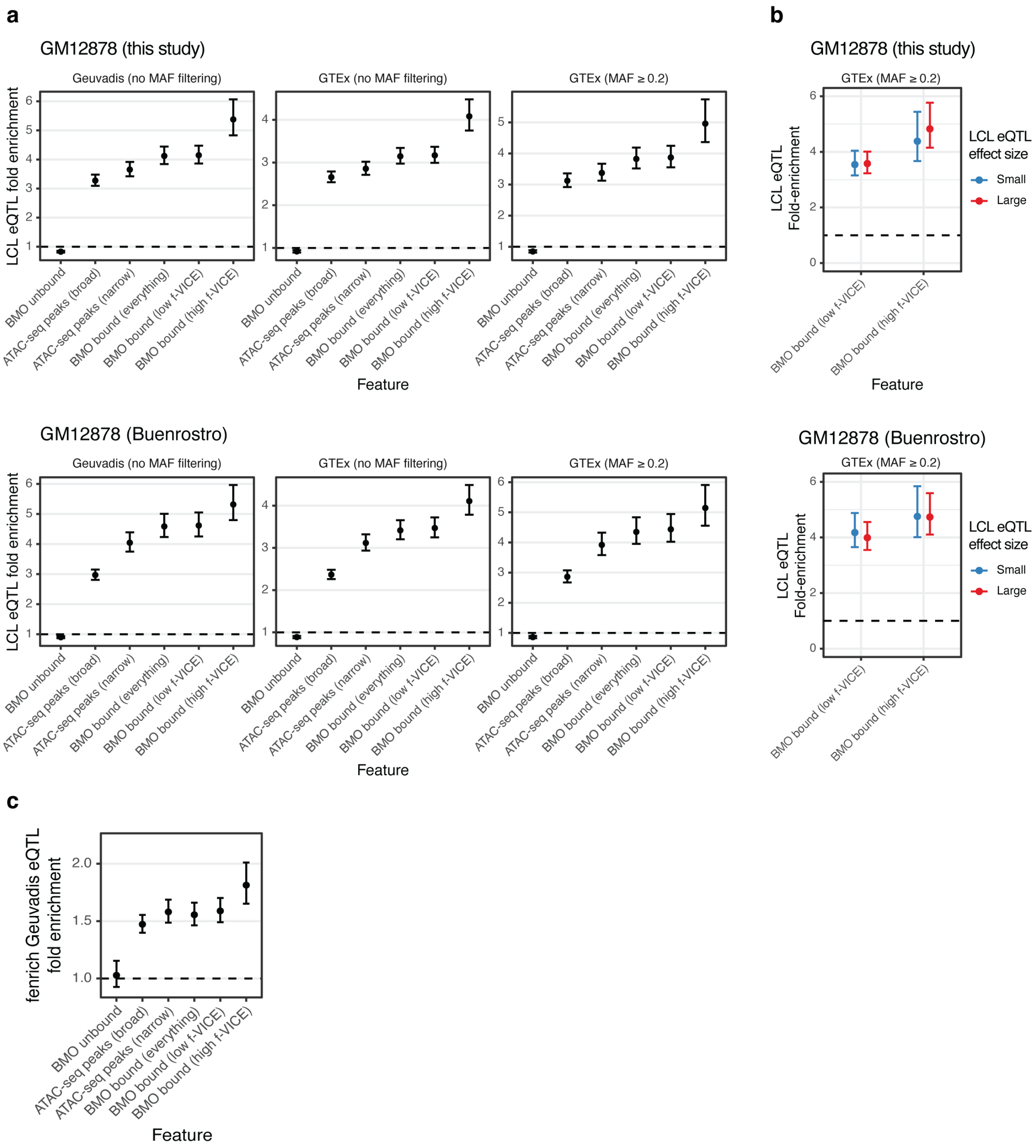

a
GM12878 (this study)
Geuvadis (no MAF filtering)
GTEx (no MAF filtering)
GTEx (MAF ≥ 0.2)
LCL eQTL fold enrichment
BMO unbound
ATAC-seq peaks (broad)
ATAC-seq peaks (narrow)
BMO bound (everything)
BMO bound (low f-VICE)
BMO bound (high f-VICE)
Feature
GM12878 (Buenrostro)
b
LCL eQTL Fold-enrichment
LCL eQTL effect size
Small
Large
c
fenrich Geuvadis eQTL fold enrichment

Supplementary Figure 24. **Enrichment of high and low f-VICE motifs in *cis*-eQTLs.**
a eQTL enrichments of different features across GM12878 ATAC-seq and lymphoblastoid cell lines (LCL) eQTL datasets. Enrichments are shown for GTEx with and without minor allele frequency (MAF) filtering to demonstrate that the observed results are not due to disproportionate representation of low MAF variants in any feature. **b** Enrichments of high and low f-VICE BMO predictions on high and low effect size GTEx eQTLs (above and below the median, respectively) across the two GM12878 datasets. **c** Geuvadis LCL eQTL enrichment calculated using QTL tools fenrich in our GM12878 dataset. Dots and error bars in all plots represent the mean standard deviation, respectively, of the effect size. Number of eQTL lead SNPs: Geuvadis (no MAF filtering) n = 2,743, GTEx (no MAF filtering) n = 3,269, GTEx (MAF ≥ 0.2) n = 1,782.

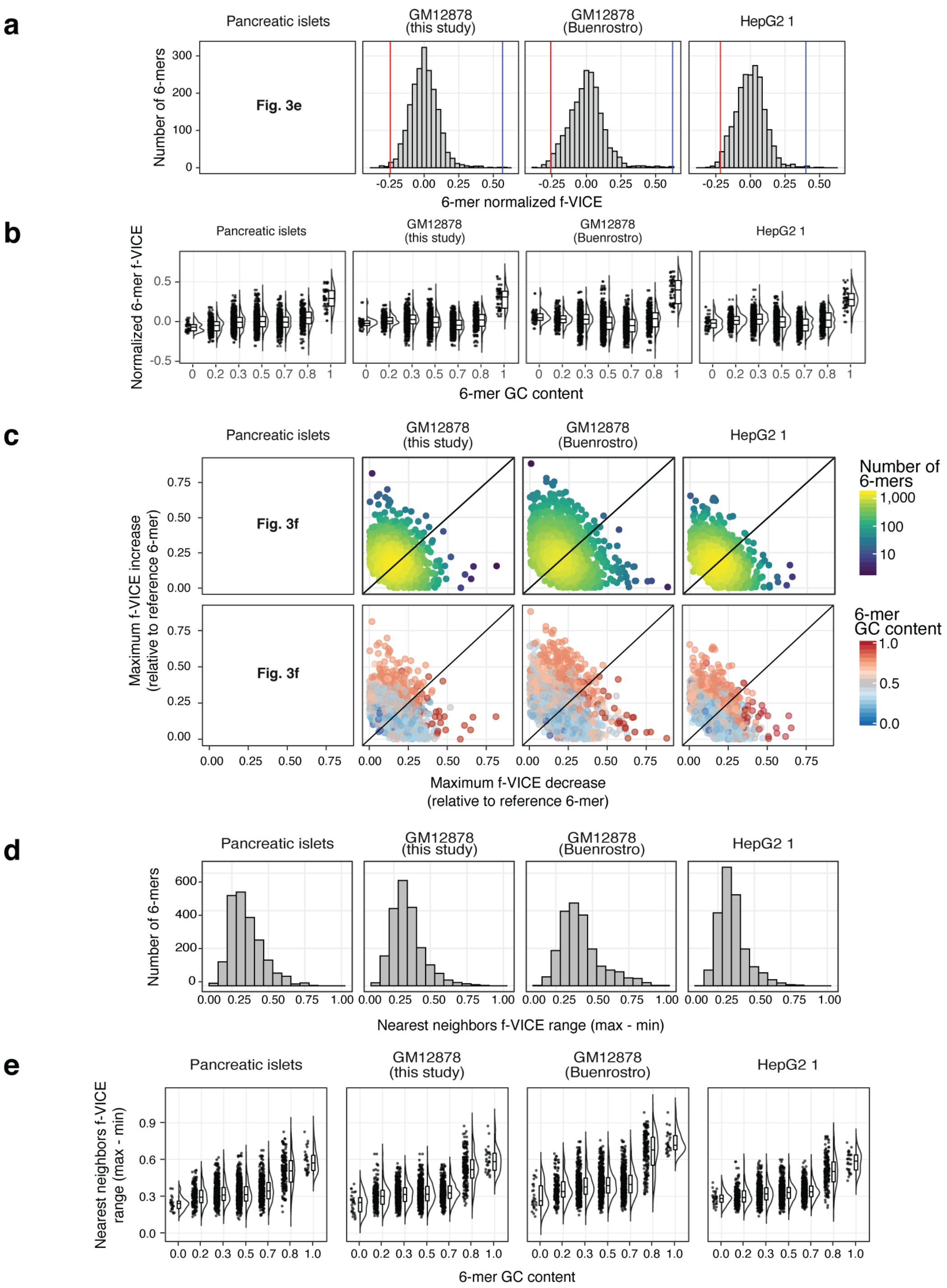

a
Pancreatic islets
GM12878 (this study)
GM12878 (Buenrostro)
HepG2 1
Number of 6-mers
Fig. 3e
6-mer normalized f-VICE
b
Normalized 6-mer f-VICE
6-mer GC content
c
Maximum f-VICE increase (relative to reference 6-mer)
Fig. 3f
Number of 6-mers
1,000
100
10
6-mer GC content
Maximum f-VICE decrease (relative to reference 6-mer)
d
Number of 6-mers
Nearest neighbors f-VICE range (max - min)
e
Nearest neighbors f-VICE range (max - min)
6-mer GC content

Supplementary Figure 25. DNA 6-mers f-VICE analyses.
a DNA 6-mers normalized f-VICE distributions across datasets. Horizontal lines represent the normalized f-IVCEs of the two 6-mers shown in Figure 3e (CGCCCC in blue and CGACCC in red). **b** 6-mer f-VICEs as a function of GC content. **c** Scatter plot of f-VICE differences for all 6-mers relative to 1bp neighbors in sequence space (*i.e.* 6-mers with a Hamming distance of 1). **d** Distribution of the f-VICE range of each 6-mer relative to its 1bp neighbors in sequence space. **e** Distribution of f-VICE range as a function of GC content. Note that high GC content 6-mers are more likely to have immediate neighbors in sequence space with lower f-VICEs. For panels **b** and **d**, boxplots centers, boxes, and whiskers represent median, 1st and 3rd quartiles, and 1.5 interquartile range, respectively. N = 2,016 6-mers in each panel facet.

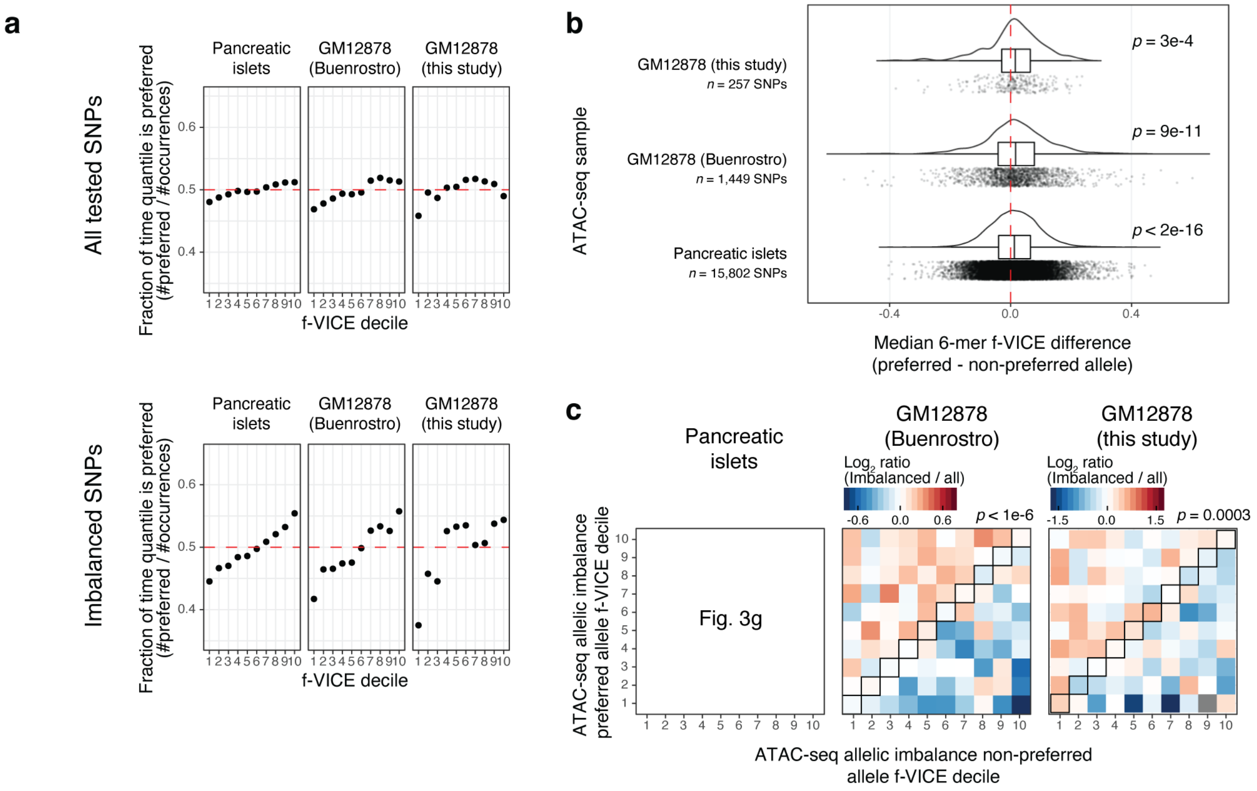

Supplementary Figure 26. f-VICE allelic imbalance analyses.
a Proportion of time the preferred ATAC-seq allele forms a 6-mer belonging to each f-VICE decile in all tested SNPs (upper) and all SNPs with significant allelic imbalance (lower). **b** Distribution of 6-mer f-VICE difference between the preferred and non-preferred alleles at loci with significant ATAC-seq imbalance. Each point corresponds to a DNA 6-mer overlapping a locus with allelic imbalance. Red dashed line corresponds to the expectation. Nominal *p*-values obtained from binomial tests (two-sided). **c** f-VICE decile transition matrices. Each square corresponds to the ratio of imbalanced versus all tested SNPs. P-values obtained from permutation tests.

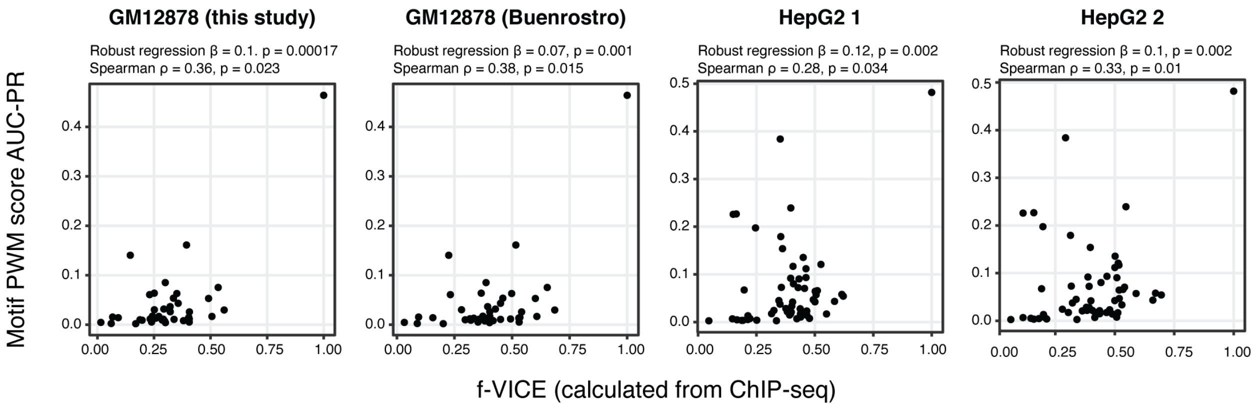

Supplementary Figure 27. f-VICE and PWM score AUC-PR.
Scatter plots of f-VICE and FIMO position weight matrix (PWM) score AUC-PR relative to ChIP-seq data data (*n* = 41 and *n* = 59 for GM12878 and HepG2, respectively). Robust linear regressions calculated using formula AUC-PR ~ f-VICE.

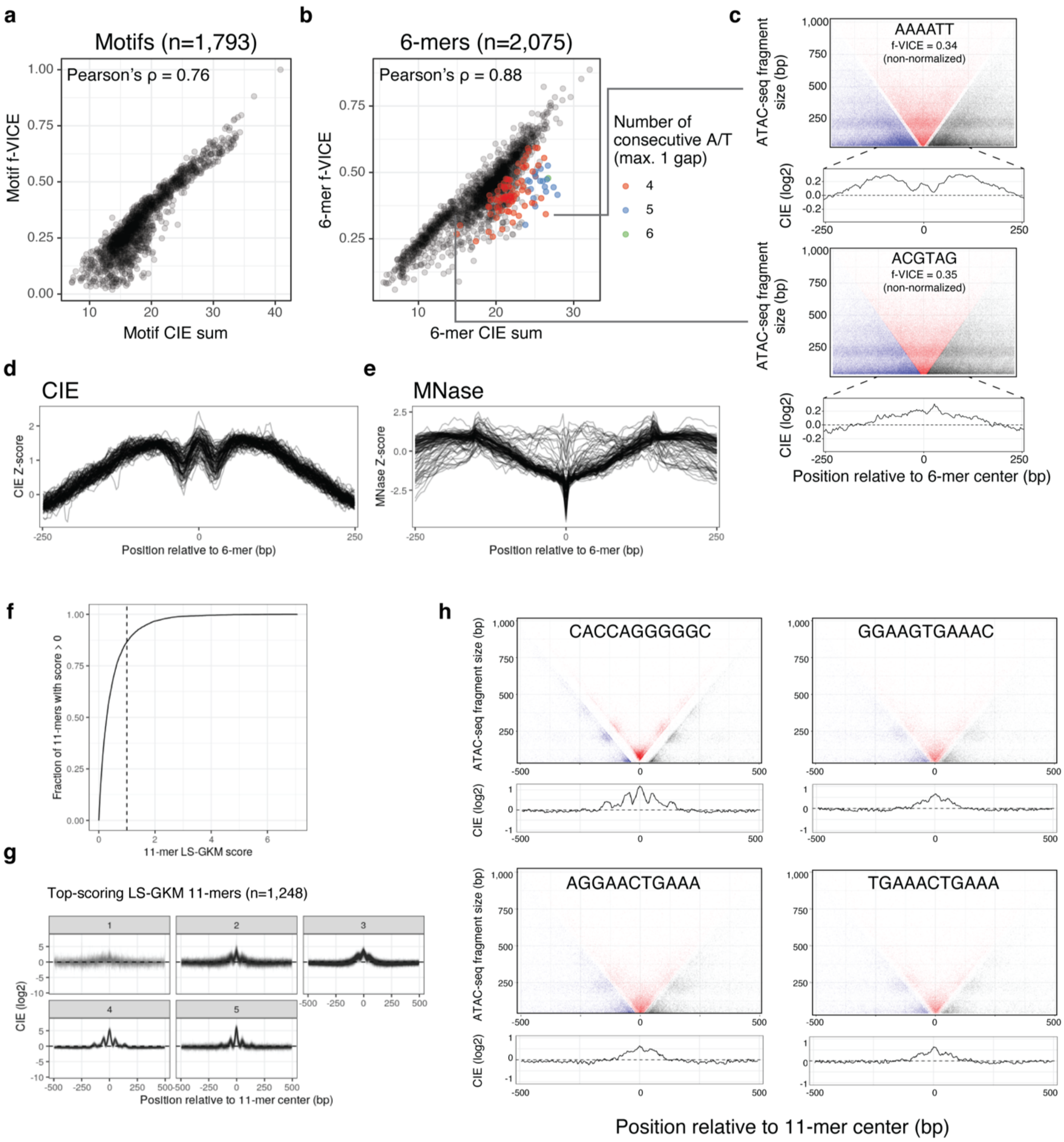

a
Motifs (n=1,793)
Pearson's ρ = 0.76
Motif f-VICE
Motif CIE sum
b
6-mers (n=2,075)
Pearson's ρ = 0.88
6-mer f-VICE
6-mer CIE sum
Number of consecutive A/T (max. 1 gap)
4
5
6
c
AAAATT
f-VICE = 0.34
(non-normalized)
ACGTAG
f-VICE = 0.35
(non-normalized)
ATAC-seq fragment size (bp)
CIE (log2)
Position relative to 6-mer center (bp)
d
CIE
CIE Z-score
Position relative to 6-mer (bp)
e
MNase
MNase Z-score
Position relative to 6-mer (bp)
f
Fraction of 11-mers with score > 0
11-mer LS-GKM score
g
Top-scoring LS-GKM 11-mers (n=1,248)
CIE (log2)
Position relative to 11-mer center (bp)
h
CACCAGGGGGC
GGAAGTGAAAC
AGGAACTGAAA
TGAAACTGAAA
ATAC-seq fragment size (bp)
CIE (log2)
Position relative to 11-mer center (bp)

Supplementary Figure 28. Additional exploration of CIE shape patterns.
a Scatter plots of f-VICEs and total (positive) CIEs from TF motifs. **b** Similar to **a**, but for DNA 6-mers. Colors represent the number of consecutive A or T nucleotides, with maximum one gap allowed (*e.g.* AAANA). **c** Upper: representative poly(dA:dT) 6-mer with a "non-canonical" CIE pattern, where there is less information at the 6-mer region compared to the 6-mer-adjacent regions. Bottom: non-poly(dA:dT) 6-mer with similar f-VICE value. **d-e** CIE and MNase patterns at the poly(dA:dT) 6-mers highlighted in **b**. **f** ECDF of LS-GKM scores > 0. Dashed vertical line represent the cutoff for high-scoring 11-mers. **g** CIE shape clustering of high-scoring LS-GKM 11-mers (n = 1,248) using k-means demonstrating that features associated with accessible DNA elements have "canonical" CIE patterns similar to the ones observed for TF motifs. **h** Representative V-plots and CIE patterns for high-scoring 11-mers.

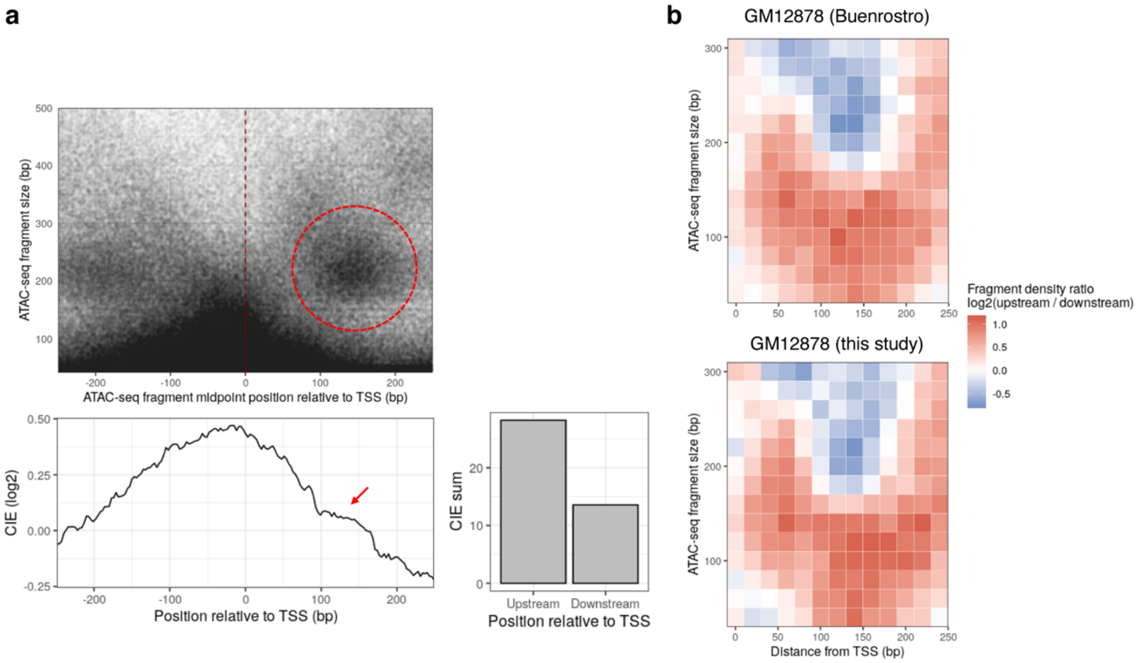

Supplementary Figure 29. CIE patterns at transcription start sites.
a ATAC-seq fragment size distribution in the vicinity of highly expressed (RNA-seq reads per million quintile 5; GTEx, EBV-transformed lymphoblastoid cells) transcription start sites (TSS) in GM12878 ATAC-seq data. The dashed red circle and red arrow indicate a well-positioned +1 nucleosome downstream of the TSS regions. The plot in the lower right quantifies the degree of CIE asymmetry between regions upstream and downstream of the TSS coordinates. ATAC-seq data: Buenrostro. **b** ATAC-seq midpoint density ratio between upstream and downstream regions relative to the TSS. The +1 nucleosome can be seen in both samples.

Supplementary Tables 1 and 2

Supplementary Table 1. FRAP recovery times from literature

Factor	Organism	Motif	FRAP recovery (s)	Reference
AHR	*Homo sapiens*	AHR_1	38	11
AP1	*Homo sapiens*	MA0476.1	600	12
ARNT	*Homo sapiens*	ARNT_2	41	11
CEBP	*Homo sapiens*	CEBPB_known5	32	11
CREB	*Homo sapiens*	CREB3_1	100	13
CTCF	*Homo sapiens*	CTCF_known2	660	14
FOXA1	*Mus musculus*	FOXA_known4	300	15
MYC	*Homo sapiens*	MYC_known13	37	11
NFKB	*Homo sapiens*	NFKB_known5	30	16
NR3C1	*Cercopithecus aethiops*	NR3C1_known18	30	17
NR3C2	*Homo sapiens*	NR3C2_1	30	18
TP53	*Homo sapiens*	TP53_4	20	19
XBP	*Homo sapiens*	XBP1_2	30	11

Supplementary Table 2. List of primers

Name	Catalog #	Fabricant	Sequence	Obs.
IndexD7XX	38096	Swift Biosciences	GATCGGAAGAGCACACGT CTGAACTCCAGTCAC[i7] ATCTCGTATGCCGTCTTC TGCTTG	[i7] indicates any 8-bp sample barcode
Kizman lab primer (1) i5 Nextera compatible	Barcode plate #10	Kitzman Lab	AATGATACGGCGACCACC GAGATCTACAC[x]TCGTC GGCAGCGTC	[x] indicates any 10-bp well-specific barcode
Kizman lab primer (2) i7 Nextera compatible	Barcode plate #5	Kitzman Lab	CAAGCAGAAGACGGCATA CGAGAT[x]GTCTCGTGGG CTCGG	[x] indicates any 10-bp well-specific barcode

Supplementary References

1. He, H. H. *et al.* Refined DNase-seq protocol and data analysis reveals intrinsic bias in transcription factor footprint identification. *Nat. Methods* **11**, 73–78 (2014).
2. Cuellar-Partida, G. *et al.* Epigenetic priors for identifying active transcription factor binding sites. *Bioinformatics* **28**, 56–62 (2012).
3. Yardımcı, G. G., Frank, C. L., Crawford, G. E. & Ohler, U. Explicit DNase sequence bias modeling enables high-resolution transcription factor footprint detection. *Nucleic Acids Res.* **42**, 11865–11878 (2014).
4. Wang, J. *et al.* Sequence features and chromatin structure around the genomic regions bound by 119 human transcription factors. *Genome Res.* **22**, 1798–1812 (2012).
5. Li, Z. *et al.* Identification of transcription factor binding sites using ATAC-seq. *Genome Biol.* **20**, 45 (2019).
6. Sung, M. H., Guertin, M. J., Baek, S. & Hager, G. L. DNase footprint signatures are dictated by factor dynamics and DNA sequence. *Mol. Cell* **56**, 275–285 (2014).
7. Sherwood, R. I. *et al.* Discovery of directional and nondirectional pioneer transcription factors by modeling DNase profile magnitude and shape. *Nat. Biotechnol.* **32**, 171–178 (2014).
8. Pique-Regi, R. *et al.* Accurate inference of transcription factor binding from DNA sequence and chromatin accessibility data. *Genome Res.* **21**, 447–455 (2011).
9. Orchard, P., Kyono, Y., Hensley, J., Kitzman, J. O. & Parker, S. C. J. Quantification, Dynamic Visualization, and Validation of Bias in ATAC-Seq Data with ataqv. *Cell Syst.* **10**, 298-306.e4 (2020).

10. Schep, A. N. *et al.* Structured nucleosome fingerprints enable high-resolution mapping of chromatin architecture within regulatory regions. *Genome Res.* **25**, 1757–1770 (2015).

11. Phair, R. D. *et al.* Global Nature of Dynamic Protein-Chromatin Interactions In Vivo: Three-Dimensional Genome Scanning and Dynamic Interaction Networks of Chromatin Proteins. *Mol. Cell. Biol.* **24**, 6393–6402 (2004).

12. Malnou, C. E. *et al.* Heterodimerization with different jun proteins controls c-Fos intranuclear dynamics and distribution. *J. Biol. Chem.* **285**, 6552–6562 (2010).

13. Mayr, B. M., Guzman, E. & Montminy, M. Glutamine rich and basic region/leucine zipper (bZIP) domains stabilize cAMP-response element-binding protein (CREB) binding to chromatin. *J. Biol. Chem.* **280**, 15103–15110 (2005).

14. Nakahashi, H. *et al.* A Genome-wide Map of CTCF Multivalency Redefines the CTCF Code. *Cell Rep.* **3**, 1678–1689 (2013).

15. Sekiya, T., Muthurajan, U. M., Luger, K., Tulin, A. V. & Zaret, K. S. Nucleosome-binding affinity as a primary determinant of the nuclear mobility of the pioneer transcription factor FoxA. *Genes Dev.* **23**, 804–809 (2009).

16. Bosisio, D. *et al.* A hyper-dynamic equilibrium between promoter-bound and nucleoplasmic dimers controls NF-κB-dependent gene activity. *EMBO J.* **25**, 798–810 (2006).

17. Groeneweg, F. L. *et al.* Quantitation of glucocorticoid receptor DNA-binding dynamics by single-molecule microscopy and FRAP. *PLoS ONE* **9**, 1–12 (2014).

18. Tirard, M., Almeida, O. F. X., Hutzler, P., Melchior, F. & Michaelidis, T. M. Sumoylation and proteasomal activity determine the transactivation properties of the mineralocorticoid receptor. *Mol. Cell. Endocrinol.* **268**, 20–29 (2007).

19. Hinow, P. *et al.* The DNA binding activity of p53 displays reaction-diffusion kinetics. *Biophys. J.* **91**, 330–342 (2006).
