# Supplementary material for: Chromatin information content landscapes inform transcription factor and DNA interactions
Source: Nat Commun. 2021 Feb 26;12:1307. doi: 10.1038/s41467-021-21534-4 (PMC7910283; doi:10.1038/s41467-021-21534-4)
Supplement: Supplementary file 3 — Description of Additional Supplementary Files [file 41467_2021_21534_MOESM3_ESM.pdf]

Description of Additional Supplementary Files

Supplementary Data 1. List of public datasets used in this study.

Supplementary Data 2. List of motif PWM clusters.

Supplementary Data 3. List of normalized and non-normalized motif and 6-mer f-VICEs for the samples analyzed in this study.
