# Supplementary material for: Chromatin information content landscapes inform transcription factor and DNA interactions
Source: Nat Commun. 2021 Feb 26;12:1307. doi: 10.1038/s41467-021-21534-4 (PMC7910283; doi:10.1038/s41467-021-21534-4)
Supplement: Supplementary file 7 — Reporting Summary [file 41467_2021_21534_MOESM7_ESM.pdf]

nature research

Corresponding author(s): Stephen C. J. Parker

Last updated by author(s): Dec 21, 2020

Reporting Summary

Nature Research wishes to improve the reproducibility of the work that we publish. This form provides structure for consistency and transparency in reporting. For further information on Nature Research policies, see our Editorial Policies and the Editorial Policy Checklist.

Statistics

For all statistical analyses, confirm that the following items are present in the figure legend, table legend, main text, or Methods section.

n/a	Confirmed	
[ ]	[x]	The exact sample size (*n*) for each experimental group/condition, given as a discrete number and unit of measurement
[ ]	[x]	A statement on whether measurements were taken from distinct samples or whether the same sample was measured repeatedly
[ ]	[x]	The statistical test(s) used AND whether they are one- or two-sided<br>*Only common tests should be described solely by name; describe more complex techniques in the Methods section.*
[ ]	[x]	A description of all covariates tested
[ ]	[x]	A description of any assumptions or corrections, such as tests of normality and adjustment for multiple comparisons
[ ]	[x]	A full description of the statistical parameters including central tendency (e.g. means) or other basic estimates (e.g. regression coefficient) AND variation (e.g. standard deviation) or associated estimates of uncertainty (e.g. confidence intervals)
[ ]	[x]	For null hypothesis testing, the test statistic (e.g. *F*, *t*, *r*) with confidence intervals, effect sizes, degrees of freedom and *P* value noted<br>*Give P values as exact values whenever suitable.*
[x]	[ ]	For Bayesian analysis, information on the choice of priors and Markov chain Monte Carlo settings
[x]	[ ]	For hierarchical and complex designs, identification of the appropriate level for tests and full reporting of outcomes
[ ]	[x]	Estimates of effect sizes (e.g. Cohen's *d*, Pearson's *r*), indicating how they were calculated

Our web collection on statistics for biologists contains articles on many of the points above.

Software and code

Policy information about availability of computer code

Data collection	HiSeq Control Software (3.3.52)
Data analysis	atactk (1.9),<br>ataqv (1.0.0),<br>bamUtil (1.0.14),<br>bedtools (2.26.0),<br>BMO (1.0),<br>BWA mem (0.7.15),<br>CENTIPEDE (1.2),<br>cta (0.1.2),<br>DNase2TF (1.0),<br>dplyr (0.7.8),<br>entropy (1.2.1),<br>FIMO (0.5.4),<br>FIMO (5.0.4),<br>fitdistrplus (1.0-11),<br>ggplot2 (3.1.0),<br>GREGOR (1.2.1),<br>HINT (0.12.1),<br>HINT-ATAC (RGT v. 1.1.1),<br>LS-GKM (commit 164a4a4),<br>MACS2 (2.1.1.20160309),

MASS (7.3-50),
mixtools (v. 1.1.0),
ngsplot (2.63),
NucleoATAC (0.3.4),
Picard (2.8.1),
PIQ (1.3),
PRROC (1.3),
Python (3.5.3),
QTL tools (1.1),
R (3.5.1),
RGT (1.1.1),
ROCR (1.0-7),
RSAT matrix-clustering (1.05),
Rstudio (1.1.456),
samtools (1.3.1 and 1.9),
Snakemake (5.5.0),
STAR (2.5.4b),
tidyr (0.8.2),
WASP (0.2.1 commit 5a52185; python version 2.7),

Custom code available at https://github.com/ParkerLab/chromatin_information.

For manuscripts utilizing custom algorithms or software that are central to the research but not yet described in published literature, software must be made available to editors and reviewers. We strongly encourage code deposition in a community repository (e.g. GitHub). See the Nature Research guidelines for submitting code & software for further information.

Data

Policy information about availability of data

All manuscripts must include a data availability statement. This statement should provide the following information, where applicable:

- Accession codes, unique identifiers, or web links for publicly available datasets
- A list of figures that have associated raw data
- A description of any restrictions on data availability

GM12878 ATAC-seq datasets generated in this study are available at GEO under accession GSE135074. Source Data file is included with this manuscript. Processed data is available at Zenodo (https://doi.org/10.5281/zenodo.3478583).
Publicly available datasets used in this study:
ATAC-seq datasets: SRX1497362, SRX1497365, SRX2717887, SRX2717888, E-MTAB-7543 (rep 3), E-MTAB-7543 (rep 1), SRX298000, SRX2768920 , SRX2768919, SRX2768918, SRX2768917, SRX298001, SRX298002, SRX298003, SRX298004, SRX298005, SRX298006
MNase-seq datasets: SRR452483
ChIP-seq datasets (GM12878): ENCFF784PEF, ENCFF794KET, ENCFF133GHG, ENCFF969FVF, ENCFF748WOQ, ENCFF006MIL, ENCFF096AKZ, ENCFF850MAC, ENCFF382VEJ, ENCFF880NTF, ENCFF476RII, ENCFF565SXH, ENCFF742XOI, ENCFF662JYS, ENCFF343VAG, ENCFF337XDI, ENCFF708VKT, ENCFF762AZG, ENCFF112CKJ, ENCFF407JNK, ENCFF288RYL, ENCFF811FYS, ENCFF006MAM, ENCFF138CXP, ENCFF861YUL, ENCFF359EFT, ENCFF269LZJ, ENCFF739VBA, ENCFF363BLT, ENCFF969EMZ, ENCFF618KHI, ENCFF936XYD, ENCFF147DQK, ENCFF040ZUY, ENCFF069KRU, ENCFF515HWO, ENCFF759PVA, ENCFF817AOQ, ENCFF294BZJ, ENCFF369JYP, ENCFF229WZB
ChIP-seq datasets (HepG2): ENCFF841GUR, ENCFF215TZZ, ENCFF510UKU, ENCFF614HYY, ENCFF502XEV, ENCFF467TLM, ENCFF419CGD, ENCFF996YOX, ENCFF329MOF, ENCFF786XDZ, ENCFF066MGX, ENCFF811CMJ, ENCFF392RQB, ENCFF653JVV, ENCFF816PYF, ENCFF860ZQP, ENCFF105NJB, ENCFF722GFS, ENCFF888ZAZ, ENCFF081YHC, ENCFF682HTY, ENCFF421ZII, ENCFF044GIB, ENCFF222YCT, ENCFF267YOX, ENCFF916SBW, ENCFF596XCU, ENCFF025GBN, ENCFF645NAR, ENCFF814UDQ, ENCFF762QHA, ENCFF754RXP, ENCFF634BXB, ENCFF266HXY, ENCFF249FDQ, ENCFF773PSQ, ENCFF487OSZ, ENCFF920ITW, ENCFF489SNI, ENCFF180BOV, ENCFF618IBW, ENCFF076BOS, ENCFF859UHG, ENCFF645MNT, ENCFF907PFI, ENCFF003NDR, ENCFF315DYP, ENCFF942TIQ, ENCFF164KYP, ENCFF810LYT, ENCFF138XKG, ENCFF263VIF, ENCFF568BTO, ENCFF834ACA, ENCFF016NASRR452483ATAC-seq datasets: SRX1497362, SRX1497365, SRX2717887, SRX2717888, E-MTAB-7543 (rep 3), E-MTAB-7543 (rep 1), SRX298000, SRX2768920 , SRX2768919, SRX2768918, SRX2768917, SRX298001, SRX298002, SRX298003, SRX298004, SRX298005, SRX298006
MNase-seq datasets: SRR452483
ChIP-seq datasets (GM12878): ENCFF784PEF, ENCFF794KET, ENCFF133GHG, ENCFF969FVF, ENCFF748WOQ, ENCFF006MIL, ENCFF096AKZ, ENCFF850MAC, ENCFF382VEJ, ENCFF880NTF, ENCFF476RII, ENCFF565SXH, ENCFF742XOI, ENCFF662JYS, ENCFF343VAG, ENCFF337XDI, ENCFF708VKT, ENCFF762AZG, ENCFF112CKJ, ENCFF407JNK, ENCFF288RYL, ENCFF811FYS, ENCFF006MAM, ENCFF138CXP, ENCFF861YUL, ENCFF359EFT, ENCFF269LZJ, ENCFF739VBA, ENCFF363BLT, ENCFF969EMZ, ENCFF618KHI, ENCFF936XYD, ENCFF147DQK, ENCFF040ZUY, ENCFF069KRU, ENCFF515HWO, ENCFF759PVA, ENCFF817AOQ, ENCFF294BZJ, ENCFF369JYP, ENCFF229WZB
ChIP-seq datasets (HepG2): ENCFF841GUR, ENCFF215TZZ, ENCFF510UKU, ENCFF614HYY, ENCFF502XEV, ENCFF467TLM, ENCFF419CGD, ENCFF996YOX, ENCFF329MOF, ENCFF786XDZ, ENCFF066MGX, ENCFF811CMJ, ENCFF392RQB, ENCFF653JVV, ENCFF816PYF, ENCFF860ZQP, ENCFF105NJB, ENCFF722GFS, ENCFF888ZAZ, ENCFF081YHC, ENCFF682HTY, ENCFF421ZII, ENCFF044GIB, ENCFF222YCT, ENCFF267YOX, ENCFF916SBW, ENCFF596XCU, ENCFF025GBN, ENCFF645NAR, ENCFF814UDQ, ENCFF762QHA, ENCFF754RXP, ENCFF634BXB, ENCFF266HXY, ENCFF249FDQ, ENCFF773PSQ, ENCFF487OSZ, ENCFF920ITW, ENCFF489SNI, ENCFF180BOV, ENCFF618IBW, ENCFF076BOS, ENCFF859UHG, ENCFF645MNT, ENCFF907PFI, ENCFF003NDR, ENCFF315DYP, ENCFF942TIQ, ENCFF164KYP, ENCFF810LYT, ENCFF138XKG, ENCFF263VIF, ENCFF568BTO, ENCFF834ACA, ENCFF016NAS, ENCFF962DEO, ENCFF858QVH, ENCFF363GGS, ENCFF004UHW

Field-specific reporting

Life sciences study design

All studies must disclose on these points even when the disclosure is negative.

Sample size	No sample size calculation was performed prior to the ATAC-seq experiments. ATAC-seq was performed using 50,000 cells as described by the Greenleaf group, and we assessed the quality of the libraries prior to sequencing using the Agilent Bioanalyzer results from the University of Michigan Sequencing Core.
Data exclusions	We excluded from the analyses publicly available ATAC-seq datasets from our survey that did not have at least 20 million high-quality autosomal reads, transcription start site (TSS) enrichment ≥ 6, and Spearman's correlation < 0.8 for the chromatin information enrichment patterns at ubiquitous and conserved CTCF-cohesin binding sites compared to the GM12878 ATAC-seq data generated in this study. We only retained ATAC-seq datasets for tissues/cells that had at least two samples that passed these criteria. Exclusion criteria were not pre-established.
Replication	Reproducibility of the ATAC-seq signal from the GM12878 dataset generated in this study was quantified by comparing chromatin accessibility levels at the union of peaks called in our sample and in the Buenrostro et al GM12878 replicate with the highest coverage (replicate 1).
Randomization	No experimental groups were defined for the analyses performed in this study. When necessary, data was grouped using unsupervised techniques (k-means clustering and hierarchical clustering).
Blinding	No blinding was performed. The chromatin information enrichment metric developed in this study was developed using our GM12878 ATAC-seq data and then applied to the remaining ATAC-seq datasets analyzed in this study.

Reporting for specific materials, systems and methods

We require information from authors about some types of materials, experimental systems and methods used in many studies. Here, indicate whether each material, system or method listed is relevant to your study. If you are not sure if a list item applies to your research, read the appropriate section before selecting a response.

Materials & experimental systems

n/a	Involved in the study	
☒	☐	Antibodies
☐	☒	Eukaryotic cell lines
☒	☐	Palaeontology and archaeology
☒	☐	Animals and other organisms
☒	☐	Human research participants
☒	☐	Clinical data
☒	☐	Dual use research of concern

Methods

n/a	Involved in the study	
☒	☐	ChIP-seq
☒	☐	Flow cytometry
☒	☐	MRI-based neuroimaging

Eukaryotic cell lines

Policy information about cell lines

Cell line source(s)	Coriell Institute for Medical Research
Authentication	No authentication due to cells being low passage (n ≤ 6)
Mycoplasma contamination	We cultured GM12878 cells with added plasmocin (Invivogen, San Diego, CA; 50 ug/mL) to the growth media to prevent mycoplasma contamination. Cells were not tested for mycoplasma contamination.
Commonly misidentified lines (See ICLAC register)	No commonly misidentified lines were used in this study.
